# Physiologically Based Biopharmaceutics Modelling—Best Scientific Practices to Define Drug Product Performance, Latest Regulatory and Industry Perspectives: Workshop Summary Report

**DOI:** 10.3390/pharmaceutics18050566

**Published:** 2026-05-01

**Authors:** Mark McAllister, Nena Mistry, Xavier Pepin, Susan Cole, Christer Tannergren, Konstantinos Stamatopoulos, Helena Engman, Andrea Moir, Chara Litou, Francesca Gavins, Sumit Arora, Maria Malamatari, Mariana Guimarães, Aishwarya Ravi, Nikoletta Fotaki, Laurence Dodd, Øyvind Holte, James Butler, Paul A. Dickinson, Matt Popkin, Andrew Butler, Orla NiOgain, Nico Holmstock, Claire Mackie

**Affiliations:** 1 Biowaived Ltd., Discovery Park, Sandwich CT13 9NJ, UK; 2 GSK R&D, David Jack Research Centre, Harris’s Lane, Ware SG12 0GX, UKkonstantinos.x.stamatopoulos@gsk.com (K.S.); james.m.butler@gsk.com (J.B.); 3 PBPK R&D, Simulations Plus, Inc., P.O. Box 12317, Research Triangle Park, NC 27709, USA; 4 Medicines & Healthcare Products Regulatory Agency, 10 South Colonnade, London E14 4PU, UKmary.malamatari@mhra.gov.uk (M.M.);; 5 Global Product Development, Pharmaceutical Technology & Development, Operations, AstraZeneca, 43183 Gothenburg, Swedenhelena.engman@sedapds.com (H.E.); 6 Global Product Development, Pharmaceutical Technology & Development, Operations, AstraZeneca, Macclesfield SK10 2NA, UK; 7 Simcyp, Certara Predictive Technologies, Certara UK Ltd., Sheffield S1 2BJ, UK; 8 Johnson & Johnson, Turnhoutseweg 32, 2340 Beerse, Belgium; 9 ESQlabs GmbH, 26683 Saterland, Germany; 10 Pfizer Healthcare India Private Limited, IITM RESEARCH PARK, Kanagam, Tharamani, Chennai 600113, Tamil Nadu, India; 11 Department of Life Sciences, University of Bath, Bath BA2 7AY, UK; n.fotaki@bath.ac.uk; 12 Norwegian Medical Products Agency, 0663 Oslo, Norway; 13 Seda Pharmaceutical Development Services, Unit 4 Oakfield Road, Cheadle Royal Business Park, Cheadle, Stockport SK8 3GX, UK; paul.dickinson@sedapds.com; 14 GSK R&D, Harris’s Lane, Ware SG12 ODJ, UK

**Keywords:** PBBM, clinically relevant dissolution specifications, dissolution safe space, best practices

## Abstract

In November 2024, a two-day meeting entitled “PBBM—Best Scientific Practices to Define Drug Product Performance: Latest Regulatory and Industry Perspectives” was organised by the Academy of Pharmaceutical Sciences Biopharmaceutics Focus group and hosted by MHRA in London, UK. Physiologically based biopharmaceutics modelling, referred to as PBBM, is used to inform drug product quality strategies and provide a more detailed understanding of how medicines can interact with the human body. Industrial, academic, regulatory, and software company scientists came together to discuss the latest developments in PBBM and to debate key topics relevant to the establishment of best practices and improved implementation. Case study presentations and breakout sessions highlighted how companies are using PBBM in their portfolio decision-making (early development through post-approval changes). Discussions highlighted how the exploration of drug product quality risks has evolved over time, moving from the more empirical BCS classification approach to a more detailed in vivo and mechanistic understanding, where sponsors have and continue to invest in building clinical drug product knowledge. Regulatory scientists shared how they are building experience in using PBBM to set clinically relevant drug product quality specifications, including how they would like to see the area grow in the future. Although significant progress has certainly been made in this field over the last 10 years, the need to continue to bring industry and regulators closer together in the future remains a key topic. Guideline evolution, training and continued dialogue will be essential in reaching a harmonised approach to the use of PBBM to develop drug product strategies and set quality specifications.

## 1. Introduction

Physiologically Based Biopharmaceutics Modelling (PBBM) has emerged as a powerful tool in pharmaceutical development, offering mechanistic insights that bridge the gap between in vitro dissolution testing and the in vivo performance of drug products. The published use case examples of PBBM across a range of applications—which span from early-stage biopharmaceutics risk assessment [1], formulation design and development [2,3,4] to supporting regulatory strategy for biowaivers and performance-led justification of dissolution specifications [5]—reflect the widespread adoption of PBBM across the different stages of the discovery and product development lifecycle. In November 2024, a two-day workshop was organised by the Academy of Pharmaceutical Sciences (APS) at the MHRA headquarters in London. This meeting attracted around 80 attendees from small, medium and large pharma companies, academia and regulatory agencies to discuss the latest developments in PBBM and to debate key topics relevant to the establishment of best practices and continued implementation. On day one, the breakout sessions covered three PBBM case study examples (previously shared at the M-CERSI meeting in August 2023) [6] from a PBBM developer’s perspective, and the discussion groups were asked to assess their adequacy in terms of the question of interest, their description of the context of use, their level of mechanistic ability to describe formulation performance, model verification/validation, and feedback (where available) from regulatory review of the models. On day two, the breakout sessions explored critical aspects associated with the development of a dissolution safe space using PBBM. This meeting report summarises the technical presentations and the discussion topics that were covered across these two days.

## 2. Day 1 Podium Presentations

### 2.1. Summary of Presentation: “An Introduction to PBBM” by Xavier Pepin, Simulations Plus

**Figure 1:**
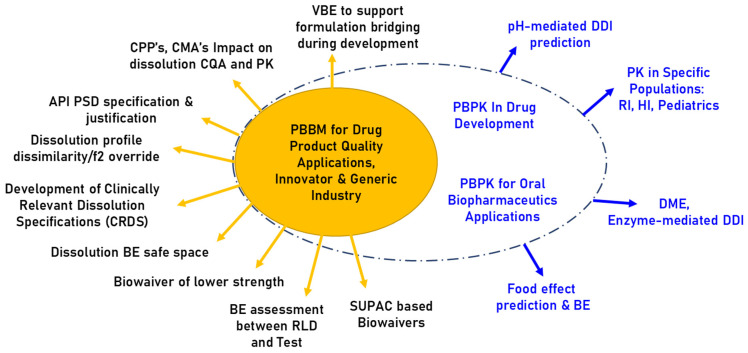
PBBM and PBPK applications for drug development.

Physiologically based Biopharmaceutics Model(s) or Modelling (PBBM) is a modelling and simulation strategy that focuses on linking drug substance (DS) and drug product (DP) quality attributes (QA) to their observed pharmacokinetics (PK) profiles. PBBM is a use case/application of physiologically based pharmacokinetic (PBPK) models where the input parameters around DS and DP have been carefully defined (Figure 1) [6].

The use of PBBM in the pharmaceutical industry is growing rapidly, as it has many benefits for understanding the limitations of drug absorption and allows scientists to improve both DS and DP characteristics. PBBM can be used throughout the drug development chain, typically with different inputs as the knowledge of the drug’s physicochemical, biopharmaceutical and pharmacokinetic attributes is generated and becomes more accurate. The late-stage applications of PBBMs can support the DS and DP control strategies, define safe spaces for critical quality attributes (CQAs) and waive bioequivalence (BE) testing for drug products beyond the dissolution similarity testing when BE cannot be waived using existing guidelines, such as the ICH M9 [7] or SUPAC guidance (Figure 1) [8,9]. The top three questions of interest (QoI) covered by regulatory submissions of PBBMs based on surveys conducted amongst the 2019 and 2022 workshop participants [2,6] are:

- Justification of DP dissolution specification;
- Justification of DS particle size distribution (PSD);
- Manufacturing site changes.

Despite the growth of PBBM use within the industry and for regulatory submissions, between half to two thirds of the submitted models are rejected by regulatory authorities [6]. The top three reasons for rejections are:

- A failed PBBM validation;
- Inadequate model parameterization;
- Clinical data for validation that did not contain non-bioequivalent (non-BE) batches.

The realisation that PBBMs are useful at all stages of development, bringing together a mechanistic understanding of DS and DP performance, can avoid unnecessary clinical trial testing to support the definition of CQAs and their acceptable ranges, and strongly encourages the interest of the scientific community, whether its members work in the regulatory authorities, academia, the pharmaceutical industry or are software-service providers in related fields. The ICH M15 guidance [10], covering model-informed drug development, provides an additional framework for PBBM, and the development of a PBBM template has helped to define minimal requirements and best practices for PBBM submissions [11]. Discussions on best practices, scientific standards, and minimum expectations for PBBM development, validation and applications started a few years ago [2,6,12,13,14,15,16,17,18], and should continue moving forward so that the acceptance rate of PBBM submissions can increase and the potential of this tool can be applied to its fullest to provide safe and effective drugs to patients worldwide.

### 2.2. Summary of Presentation: “PBBM and MIDD—A Wider Review of Regulatory Guidance and Positioning” by Sue Cole, MHRA & Flora Musuamba Tshinanu, AFMPS—FAGG

Sue Cole (MHRA) presented on past regulatory guidance from 2015 onwards, which outlines expectations from both the EMA and FDA on the reporting and evaluation of PBPK models in regulatory submissions:

- EMA guideline on the pharmacokinetic and clinical evaluation of modified release dosage forms [19].
- EMA guideline on: “Guideline on the reporting of physiologically based pharmacokinetic (PBPK) modelling and simulation” [20].
- FDA guidance on: “Physiologically Based Pharmacokinetic Analyses—Format and Content Guidance for Industry” [21].
- The Use of Physiologically Based Pharmacokinetic Analyses—Biopharmaceutics Applications for Oral Drug Product Development, Manufacturing Changes, and Controls [22].

Adequate evaluation of models used in regulatory submissions is a very important aspect influencing model acceptance. Although there is a lot of consistency in the requirements, there has been some confusion generated over the use of different terms in the guidance around the validation of methods, e.g., validation, verification and qualification. In the EMA PBPK guidance [20], comprehensive guidance was given in the form of two appendices on the qualification of the PBPK platform and the evaluation of the predictive performance of a drug model. The term Regulatory Impact was also used. This impact term has been used by European regulators in relation to the evaluation of models over the last decade and is linked to (i) the risk to the patient if the modelling predictions or assumptions lead to erroneous regulatory decisions and (ii) how much weight the evidence from the PBPK simulation will have in a certain scenario, such as the therapeutic context, and the resulting treatment recommendations. High-impact scenarios are those where the model has a high level of influence on the regulatory decision, e.g., when used in place of a clinical study to inform medicine use on the drug label. This would require a high level of qualification of the model.

The term qualification in this context relates to the consideration of whether there is enough scientific support for a certain use of a model and is therefore dependent on model use. It is described as the “*ability to predict a large dataset of independent data thereby showing the platforms ability to predict a certain purpose.*” Some details were given in terms of expectations for the qualification dataset: this should include compounds with similar ADME characteristics to those of the intended use. A range of PK characteristics that could influence the outcome should be covered, and a number ranging from 8 to 10 was suggested as a rough guide.

Assessment of the predictability of the drug model is described as: “*The process of establishing confidence in the drug model. The reliability is assessed on the basis of how well important characteristics of the drug model has been tested against* in vivo *PK data and whether adequate sensitivity and uncertainty analyses have been conducted to support the model’s ability to provide reliable predictions.*” Guidance is also given on the dataset to be used in terms of the range of different doses and conditions that must be included, the parameters to be considered, and the accuracy of prediction. The importance of addressing uncertainty in the model by way of a sensitivity analysis is also discussed.

A harmonised approach to model evaluation is required. At the FDA PBPK Workshop in Washington in 2019, in a presentation entitled “Development of Best Practices in Physiologically Based Pharmacokinetic Modeling to Support Clinical Pharmacology Regulatory Decision” [23], Colleen Kuemmel (FDA) introduced a risk-based credibility assessment framework for Model-Informed Drug Development and proposed its potential application to physiologically based pharmacokinetic modelling and simulation.

Flora Musuamba Tshinanu (AFMPS—FAGG) presented the experience of the EMA concerning applications of mechanistic models (e.g., PBPK and QSP models) to drug development, including study design optimisation for adult first in human (FIH) trials, Phase 2 and Phase 3 studies, dose selection for paediatric studies, paediatric dose characterisation for labelling (waiver of efficacy/safety studies in the target population), assessment of DDI (waiver of dedicated DDI study), formulation bridging, bridging modes of drug administration, and supporting manufacturing process changes (waiver of PK comparability study). High variability and inconsistencies in the approaches, data and methodologies proposed by applicants were noted. A Q&A is being finalised, and an EMA concept paper is planned to be published on this topic. The EMA considered there to be a need for a framework for the evaluation of mechanistic models in regulatory submissions to enable regulatory bodies to efficiently perform adequate assessments. This would include technical standards for data/evidence generation, analysis and reporting. Such standards should be acknowledged by all stakeholders, i.e., regulators, patients, academia, industry, health technology assessment (HTA) agencies, and healthcare professionals.

An ICH group composed of representatives of industry and regulators across the globe, initially led by Scott Marshall (GSK) and now by Kristin Karlsson (MPA), has recently produced the first draft of this guidance, the M15 guideline on general principles for model-informed drug development [24]. Comments have been received, and the final adoption is planned for the fourth quarter of 2025.

**Figure 2:**
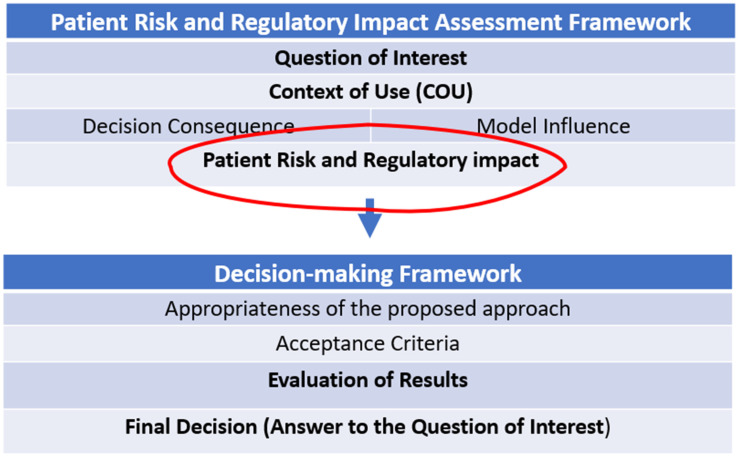
The risk-based approach to model evaluation and credibility assessment used by ICH MI15.

The guidance advocates the use of a risk-based approach to model evaluation (Figure 2). This means that the scrutiny and questioning from regulators during model assessment should always take into account: (1) the context of use, (2) the role/place of the model in the totality of evidence that supports the answer to the relevant question, (3) the consequences of an adverse outcome resulting from an incorrect decision that was made based on the model and (4) the benchmarking of the model against what would traditionally have been done (or accepted by regulators) to address the relevant question without modelling.

All perspectives need to be considered, i.e., those of the developer, patient, prescriber and regulator. The impact of the model should also be included in the regulatory submission. The model should be benchmarked against alternative sources of evidence, and alternative approaches need to be considered.

Technical standards are important for facilitating data and knowledge flow across development and for uncertainty mitigation. These include standards for data searching, structuring, formatting, and analysis, and should be considered within an attributes framework.

Flora concluded her talk with two take-home messages: (1) There is still an unmet need for regulatory guidance, including technical standards for assessment of mechanistic models, and (2) these technical standards should be informed by a risk-based analysis and a question-centric approach.

### 2.3. Summary of Presentation: “Model Build and Evolution—Developing a Model for Formulation Guidance and Biopharmaceutics Risk Assessment” by Christer Tannergren, AZ

This presentation investigated key considerations in initial PBBM development and its applications in early biopharmaceutics developability risk assessments, as well as in early drug product design/development.

The merits and gaps of the Biopharmaceutics Classification System (BCS), the Developability Classification System (DCS), and PBBM in biopharmaceutics developability risk assessments were presented. The BCS is very useful for identifying the absorption rate-limiting factor(s) of a drug; however, because of its original intended use, it is rather conservative in nature and lacks the ability to provide a quantitative assessment of the extent of absorption [25]. DCS was designed for developability applications, but it considers key biopharmaceutics properties in isolation [26]. In contrast, PBBM can account for the combined effects of gastrointestinal physiology, the physicochemical/biopharmaceutical properties of the drug, and formulation aspects to produce a quantitative prediction of absorption and exposure, making it a valuable risk-assessment tool. Indeed, PBBM-based biopharmaceutical candidate drug risk assessments have been used within AstraZeneca since 2007. Within early development, PBBM applications include assessment of the possibility of achieving therapeutic plasma exposure, predictions of dose and particle size dependency, setting the initial in vitro dissolution target and predicting the in vivo performance of early formulation prototypes, including early bridging-risk assessment [27,28].

The development of the initial PBBM, which is often used during lead optimisation, is based on bottom-up modelling approaches, as clinical pharmacokinetic data are lacking at this stage of development. Similarly, as minimal formulation or in vitro dissolution data are available, the initial model relies on software-specific theoretical dissolution equations or virtual dissolution profiles as input. Also, the default settings for the physiology parameters are usually initially applied. The importance of generating standardised high-quality physicochemical and biopharmaceutics input data, and the use of models that have been properly evaluated in relation to their intended purpose using an external dataset, was emphasised. A model prediction performance of absolute average prediction error (AAPE) ≤ 50% or absolute average fold error (AAFE) between 1.25 and 2 is sufficient to predict absorption for candidate drug selection and for early drug product design purposes [29,30]. Successful examples of such model evaluations were presented, highlighting the applications of an early PBBM [29]. These included conducting biopharmaceutical candidate drug risk assessments by predicting the fraction absorbed (Fabs) and maximum absorbable dose (MAD), establishing a theoretical dissolution safe space to define early dissolution targets in product design, supporting early particle size decisions, and predicting the regional/colon absorption and in vivo performance of extended release (ER) drug candidates.

The initial PBBM may be updated several times during the drug product development process as new in vitro and clinical pharmacokinetic data on different formulations emerge. As the PBBM is revised, the modelling approach will switch to a top-down approach. Because of the change in modelling approach and the potentially increased impact of the decisions being made based on the modelling result, the prediction performance requirements also increase when the model is revised. The final PBBM would be based on appropriate in vitro data, as well as on pharmacokinetic data from the final drug product and drug product variants, using identified critical bioavailability attributes (CBAs), critical process parameters (CPPs) and critical material attributes (CMAs), as appropriate. The final PBBM also needs to be fully validated and typically demonstrate a prediction performance of AAPE ≤ 20% or AAFE ≤ 1.25 before the PBBM may be used for regulatory applications [11,22].

### 2.4. Summary of Presentation: “Approaches to Input Formulation Properties to PBBM, Solid Form/Formulation Selection and Development” by Konstantinos Stamatopoulos, GSK

A strategy was described to combine Simcyp™ v22 (Certara Ltd., Sheffield, UK) and DDDplus™ (Simulation Plus, Inc., Research Triangle Park, NC, USA) to input formulation properties for drug product development for a BCS IV zwitterionic API (GSK3640254).

In vitro solubility and permeability data were integrated into a PBBM (Simcyp™ v22) to predict the food effect observed in clinical studies [31]. The model was developed and verified using clinical data from an immediate release (IR) tablet (10–320 mg) obtained in Healthy Volunteers (HVs) under fasting and fed conditions. Biorelevant media, containing oleic acid and cholesterol at levels representing fasted or fed states, enabled the model to appropriately capture the magnitude of the food effect. The in vitro permeability, determined using MDCK cell lines, showed that GSK3640254 permeability was enhanced by the presence of mixed micelles. The verified PBBM was then applied to define a clinically relevant dissolution safe space using criteria relevant to the target efficacious concentration of the drug at 24 h post dose (C_24h_). The PBBM suggested that ≥70% of the drug should be dissolved within 1 h to ensure no impact on the efficacy of the drug, and this was used as the lower end of the safe space. The safe space was further verified using predictive quality control (QC)/biorelevant dissolution profiles from capsules and tablets compared in relative bioavailability (rBA) studies [32]. The TNO gastrointestinal model 1 (TIM-1) system was also used to compare over-granulated batches with the reference IR tablet to further verify the safe space derived from the PBBM.

The DDDplus™ model was developed for the control of the IR tablet for a fixed granulation size and validated against batches with a range of disintegration times produced at various compression forces. The model was able to predict the impact of disintegration time on the dissolution profile of the tablet. The model was used to understand how tablet tensile strength, which is changed by compression force, affects the dissolution rate of the tablet, and the PBBM-derived safe space was used to inform the process control strategy.

The model strategy presented can be effectively adopted to increase confidence in using PBBMs to predict the food effects of BCS IV drugs. Moreover, this approach illustrates how to couple predictive dissolution models (e.g., DDDplus™) and PBPK platforms (e.g., Simcyp™) to link formulation characteristics to in vivo performance.

### 2.5. Summary of Presentation: “Regulatory Applications for Formulation Development—Particle Size and Dissolution Specification” by Claire Mackie, Johnson & Johnson

Within drug product development, drug product specifications ensure that the product remains consistent in terms of performance and quality. This is achieved through high-quality design and identifying acceptable ranges by understanding the CQAs, which include CMAs and CPPs. The desired specification should, therefore, (1) be as broad as possible, while ensuring consistent quality, (2) aim for clinical relevance, where a link can be made to the PK to demonstrate that variations within a certain range do not impact the rate and extent of absorption, (3) facilitate easier manufacturing and (4) reduce regulatory burden by demonstrating that the quality of the product remains consistent within the defined range, avoiding extensive post approval changes.

PBBM is a powerful tool that can be used to set specifications, with the prerequisite that the question of interest and context of use of the model must be defined in the model’s development, as well as its verification, validation and subsequent application. Two examples were presented to demonstrate how PBBM had been used in specification setting at J&J.

In the first example, PBBM was used to assess the impact of DS PSD on the exposure of JNJ-X, which is a BCS II API with high permeability and low solubility in all aqueous media. A PBBM was built to identify the manufacturing space for the DS and to position future DS PSD specifications. Model development used all available drug substance and drug product data, and model validation was performed using a rich independent clinical trial dataset. Simulated mean profiles compared to the observed plasma concentration time profiles indicated that the shape and variability in concentration–time profiles were captured well, together with the food effect and DS particle size across the suggested clinical dose range. The model also highlighted that C_max_ in the fed state is mainly influenced by the gastric residence time rather than drug dissolution for the smaller DS PSD formulations.

PBBM was used to identify this manufacturing space with the aim of setting up a future starting point for the DS PSD specifications. The initial DS had a medium dv50. The team was interested in a coarser DS for process capability flexibility, and therefore, a risk assessment was performed, looking at the interplay between DS dv50 and mean gastric residence time. The sensitivity analysis demonstrated that C_max_ is very sensitive to gastric emptying, whereas AUC is not very sensitive to gastric emptying, and that above a certain dv50 particle size, a drop in exposure may take place. In conclusion, the interplay between dose, prandial state, DS PSD and impact on C_max_/AUC should be considered.

**Figure 3:**
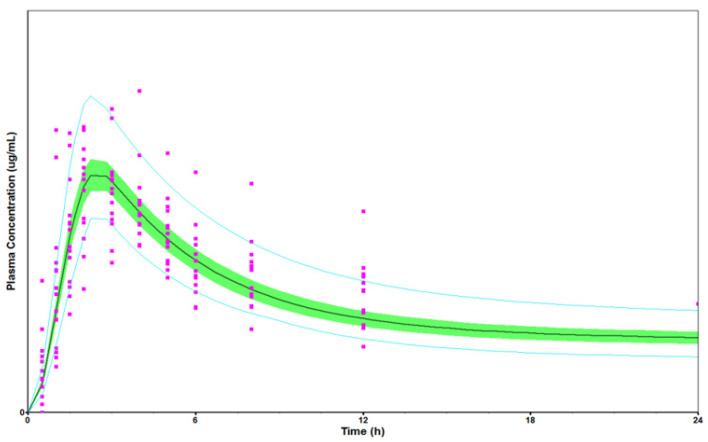
Virtual population simulation (blue lines) compared to the plasma concentrations (pink points) from one of the relative bioavailability studies.

In the second example, a PBBM was developed to set clinically relevant dissolution specifications (CRDS) for JNJ-Y—which is also a BCS II DS with high permeability and low solubility and is a neutral species across the physiological pH range—with an IR tablet for QD dosing. The next step was to build and validate the PBBM. Dissolution data were integrated into the model using the Z factor approach. The model was verified against a clinical relative bioavailability dataset, which included different formulation platforms and various process scale-up batches that varied both CMAs and CPPs. Figure 3 shows an example of a virtual population simulation compared to the plasma concentrations from one of the studies.

Two important CQAs were identified, for which both the QC dissolution and physiologically based dissolution testing (PBDT) methods could pick up the variations. Variations in CQA 1 (particle size distribution) were tested in a relative bioavailability study; the PK was comparable, and the model predicted the same outcome. For CQA 2 (polymorphic purity), PBDT was used as an input to PBBM, with VBE used to evaluate the impact of variations. VBE simulations showed that if CQA 2 was maintained below a certain amount, VBE trials (*n* = 10) would achieve the BE criteria for both C_max_ and AUC. For the dissolution specification setting, the approach utilised a combination of the in vivo relative bioavailability outcome and VBE to define a safe space. The specification suggested was Q = 80% at 30 min. This specification would also discriminate against batches that were non-BE based on the VBE simulation outcome and, in effect, provide a space to operate in. This specification has been proposed and accepted in many countries. The PBBM for CQA 2 was one of the case studies at the 2023 workshop [6,17].

In conclusion, the examples highlighted how PBBM can play a pivotal role in drug product specification setting by enhancing CQA understanding along with in vivo drug product performance using a scientific and risk-based approach.

### 2.6. Summary of Presentation: “FDA Biopharmaceutics Experience with PBBM Guidance and PBBM Submissions” Presented by Hailing Zhang, FDA and Reported by Mark McAllister

This talk introduced patient-centric quality standards as a potential route to increase flexibility while maintaining quality by establishing acceptance criteria based on clinical performance rather than process capability or manufacturing process control, and thereby avoiding specifications that are under- or over-discriminating and not in the best interests of the patient. A major obstacle to establishing patient-centric quality standards is that the link between in vitro and in vivo performance is either missing or weak, as dissolution often lacks biorelevance and/or is not biopredictive. It was also suggested that a comprehensive evaluation of all CBAs for a formulation in clinical bioavailability studies is impractical and expensive. PBBM was suggested to provide the crucial link between in vitro and in vivo performance to establish patient-centric quality standards, assuming that model development and validation are performed appropriately. In this context, PBBM can be applied to develop patient-centric quality standards for dissolution methods and acceptance criteria by establishing a dissolution safe space. PBBM can also be used to inform CMAs and process parameters that can affect bioavailability (CBAs), such as particle size distribution and polymorphism. It can also be used to provide supportive evidence for biowaivers through virtual bioequivalence studies and assist with the justification of SUPAC level 3 changes or additional strengths. It was proposed that model-based alternative bioequivalent approaches may be a future possibility. PBBM applications in the pharmaceutical quality area need to be assessed in terms of overall risk against the totality of data available and the knowledge base available. Lower risk scenarios are likely when a larger dataset and an established knowledge base are available, for example, when setting acceptance criteria. Higher risk scenarios occur when there are multiple unknowns and there is a large impact associated with the model output, e.g., justification for a full biowaiver request. A snapshot of the FDA’s experience with PBBM was provided, with an estimated 50 A/NDA and IND submissions using PBPK to support biopharmaceutics. Currently, all PBPK modelling and simulation data to support biopharmaceutics are assessed as part of the regulatory submission, and from this dataset, 48% were found to be acceptable. Guidance continues to develop, and further progress beyond the 2020 FDA draft guidance is likely in the future. Prior to the publication of the draft guidance, approximately 35% of submissions related to dissolution specification, 35% to particle size distribution specification, 25% to general risk assessment, and just 5% focused on SUPAC changes. Following the publication of the draft guidance, there was a significant uptick in submissions using PBBM to support SUPAC changes (35%), with dissolution specifications (37%) and particle size distribution specifications (18%) still representing key focus areas. Dr Zhang suggested that improvements in submissions following the publication included the use of dedicated modelling reports, clear definitions of model purpose, scientifically justified selections of dissolution model and the provision of model and support files for review. Three case studies were briefly reviewed—(1) an example where the final drug substance particle size specification was selected based on parameter sensitivity analysis and data from virtual bioequivalence studies, (2) an example where PBBM was used to predict the impact of batches not meeting the current dissolution specification and explore the edge of failure and (3) an example where a virtual batch with a 25% slower release rate than the pivotal test formulation was applied to justify the dissolution specification for an IR product. In summary, the challenges for PBBM in regulatory submissions include instances when sparse in vivo data are available for model verification or when there are constraints associated with model validation. The presentation concluded with an update on FDA initiatives to support modelling simulations, which include a model-informed drug development paired meeting programme (MIDD), a model-integrated evidence (MIE) meeting pilot programme and model master files (MMF). Further information was provided on the CDER quantitative medicine centre of excellence (established in March 2024), which is a CDER-wide enterprise that will facilitate the continuous evolution and consistent application of quantitative medicine for drug development and regulatory decision-making.

### 2.7. Summary of Presentation: “IQ/AAM Working Group—Biopharmaceutics Risk Assessment Initiative” by Helena Engman, AZ

A Critical Bioavailability Attribute (CBA) is defined by the FDA as a formulation or process variable that is expected to critically impact the bioavailability of a drug substance. The FDA has proposed a strategy emphasising the role of dissolution testing in controlling CBAs that focuses on the assessment of the bioavailability/bioequivalence risk associated with a product. The risk level provides guidance on how much effort should be spent on patient-centric/clinically relevant dissolution specifications to mitigate that risk.

A combined IQ (International Consortium for Innovation and Quality in Pharmaceutical Development) and AAM (Association for Accessible Medicines) group has, in response to a request from the FDA for feedback, built on this strategy by applying ICH Q8/Q9 principles in the context of drug product development to (1) clarify the role of dissolution testing in the assessment of product quality and manufacturing changes with varying levels of risk, (2) propose a tool for in vivo understanding to be applied at different levels of risk and formulation, (3) outline CBA identification to support robust risk mitigation and (4) define what a meaningful change is for medium-risk compounds/products.

Biopharmaceutics’ bridging risk levels are stratified based on the BCS and a mechanistic understanding of drug absorption. For BCS 1 and 3 (Very Low to Low Risk), high-solubility drugs rely on in vitro dissolution tests following established guidance, with biowaivers often being applicable. Material and process attributes are monitored to ensure acceptable dissolution performance. For BCS 2 and 4 (Medium to High Risk), drugs with absorption-limiting factors that are mechanistically understood, risk management and mitigation research utilises dissolution studies, PBBM, and existing clinical data to establish robust control strategies. In cases where mechanistic understanding is lacking, clinical data from a dedicated study, supporting IVIVR and/or PBBM, are needed to justify acceptable ranges for CBAs, and broader controls need to be implemented to mitigate risks.

The iterative process of identifying and confirming an actual CBA from a potential CBA (pCBA) follows a tiered approach, going from confirmation of in vitro relevance to confirmation of in vivo relevance. For medium- and high-risk products, further investigations may be needed for clarification. An actual CBA needs to be controlled through appropriate assays and controls to prevent meaningful changes in drug product performance. A pCBA is removed from consideration if it cannot be confirmed.

A meaningful change in either material or process attributes is a change that is likely to result in a significant change in bioavailability/bioequivalence. This highlights the industry’s consensus that dissolution methods should demonstrate sensitivity to meaningful changes in material or process attributes rather than arbitrary variations. This will ensure that the dissolution method reflects changes of clinical relevance. Two scenarios are provided for illustration:

Scenario A: The method shows an appropriate sensitivity, and changes in the process/material attributes can be assessed and controlled. A negative impact on clinical performance can be avoided. Dissolution limits absorption.

Scenario B: The method is likely overly discriminative; changes resulting in dissimilar dissolution profiles are evaluated in the context of SUPAC. Meaningful changes are relative to clinical batch experience. Dissolution does not limit absorption.

In conclusion, this summary captures the key elements of the innovator and generic industries’ approaches to the identification and control of CBAs through dissolution testing and biopharmaceutics risk management, emphasising the integration of in vitro and in vivo data, mechanistic modelling, and regulatory frameworks to ensure robust product quality and performance.

The next step in the FDA/IQ/AAM initiative is a 2-day workshop at MCERSI in 2026 to further discuss the role of dissolution testing in identifying and controlling CBAs for IR and MR solid oral dosage forms. The workshop will cover various approaches used by the innovator and generic industries to justify the selection of in vitro dissolution methods for assessing product quality at release and mitigating biopharmaceutical risks in response to changes in CBAs.

### 2.8. Day One Breakout Summaries and Discussion

#### 2.8.1. “PBBM to Support Dissolution and Particle Size Specifications” by Andrea Moir, AstraZeneca

**Figure 4:**
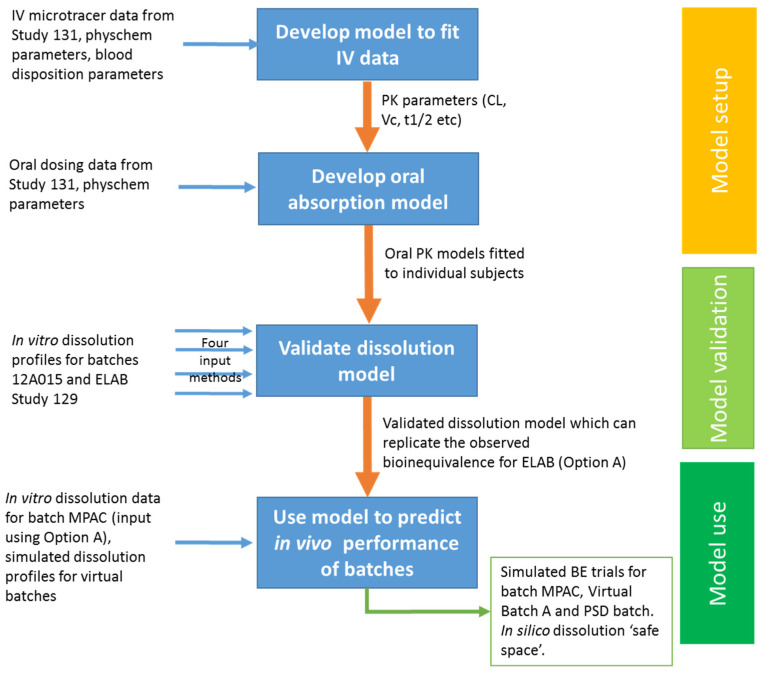
PBPK modelling strategy.

This case study considers the use of PBBM to justify the dissolution and drug substance particle size specifications for lesinurad immediate release tablets [33]. The model was built specifically in response to a request from the FDA to demonstrate, via in silico predictions or an in vivo study, that a tablet batch with dissolution at the limit of a Q = 80% in 30 min specification (batch reference MPAC) would give similar in vivo performance to that of a typical clinical tablet batch (12A015). To meet the required timelines, the model had to be built, validated, applied and reported in approximately two weeks; this was made possible by the availability of IV PK data as well as dissolution and oral PK data for batches 12A015, MPAC and a non-BE batch ELAB. The modelling strategy that was developed is summarised in Figure 4.

For model building, a “top-down” approach consisted of fitting individual disposition models to the IV PK data for the 10 subjects in Study 131. Individual subject data were used due to the observation of multiple peaks in the oral PK profiles for three of the subjects, making the determination of a meaningful mean Cmax challenging. Multiple peaks for other acidic drugs have been reported by Li et al. for non-steroidal anti-inflammatory drugs [34]. The multiple peaks were accommodated in the model by using the mixed multiple dose functionality in GastroPlus to reproduce the observed multiphasic gastric emptying of lesinurad. Lag times were also observed for many of the subjects. The measured lag times were applied directly to the model as an input to the gastric emptying phases.

To optimise the model, the small intestine and colon fluid volumes were reduced from 40% and 10% to 7.5% and 2%, respectively, for every subject; this is supported by parameter sensitivity analysis and the literature [35,36]. Effective jejunal permeability Peff was optimised for each subject with an average fitted Peff of 2.9 × 10^−4^ cm/s compared to a measured value of 3.0 × 10^−4^ cm/s. The RSD of the average fitted individual Peff values was +/−35%, which is well within the range of 16% to 125% reported by Sugano et al. for human Peff values determined across eight compounds [37].

Batch-specific dissolution data were incorporated into the model following the evaluation of four approaches; these were (1) P-PSD with the dosage form set as “Delayed Release Tablet Enteric Coated”, (2) Weibull function with the dosage form set as “Controlled Release Dissolved” or (3) “Controlled Release Undissolved” and (4) Z-factor with the dosage form set to “Delayed Release Tablet Enteric Coated”. Of these four approaches, only the P-PSD enabled the bio-inequivalence of ELAB vs. 12A015 to be reproduced. The delayed-release, enteric-coated tablet was selected in combination with the P-PSD input to ensure that the drug did not start to dissolve in the stomach, as solubility in acidic media is negligible. This was one of the first applications of P-PSD, and after approximately 10 years’ experience with this approach, the suggested current best practice would be to first select an IR tablet in the model to determine whether the P-PSD, in combination with the input solubility data, would restrict solubilisation in the gastric compartment.

Virtual clinical trials were run in a cross-over design with *n* = 25 healthy subjects to compare MPAC and ELAB to 12A015. Default parameter ranges were employed, except for stomach pH and gastric transit time, for which values were randomly assigned to each subject within the ranges of 1–4 for gastric pH and 0.1–3 h for gastric residence time. The geomean AUC_(0–96)_ and Cmax ratios for ELAB vs. 12A015 were comparable to measured data, and the ratios for MPAC vs. 012A015 were close to one.

Even though no significant difference in in vivo performance was predicted for MPAC vs. 12A015, to further support the dissolution safe space, a P-PSD was fitted for Virtual Batch A with a dissolution profile between those of MPAC and ELAB. A virtual trial was run for this virtual batch, and again, the geometric mean ratios for AUC and Cmax were close to 1. Whilst the 90% confidence intervals were tighter than might be expected for a clinical study, the virtual trial outcomes indicate that both MPAC and Virtual Batch A are likely to be bioequivalent to 12A015 in an appropriately sized study, thereby supporting the proposed dissolution specification.

#### 2.8.2. Breakout Group Discussion

After the presentation, the breakout session was facilitated by Claire Mackie (J&J) with Chara Litou (Certara) and Francesca Gavins (MHRA) as scribes. The group discussed the case study through the evaluation of three key questions:

**Q1: Were the Question of Interest (QoI) and Context of Use (CoU) for the PBBM sufficiently clear?**

The group identified two key QoIs: the dissolution specifications and DS PSD specifications. The dissolution specifications were clear, whereas the DS PSD specifications were less well-defined.

In terms of the CoU, regulatory representatives agreed that the question was addressed clearly. It was noted that this submission was accepted by EMA based on dissolution data, without requiring submission of the PBBM model. It was acknowledged that the work was completed in one week due to regulatory timelines, and it is an older case study, so the strategy may differ today.

**Q2: Was the PBBM sufficiently (mechanistically) descriptive and verified/validated?**

The group engaged in a general discussion on model inputs and the handling of dissolution and DS PSD data in model development. In effect, questions two and three were combined. The DS PSD model input and the applicant’s approach to addressing the DS PSD specifications request were challenged. For this PBBM, the applicant calculated the P-PSD from dissolution data rather than the DS PSD data, measured by laser diffraction. The group agreed that DS laser diffraction data should not be used in future models; instead, the DS PSD in the drug product should be used. It was concluded that the totality of the data should be considered, particularly given the high solubility of the DS in the intestinal segment. To address the first QoI (dissolution specifications), the P-PSD approach was followed, fitting the DP PSD to the dissolution data and not using the DS PSD measurements. However, to address the second QoI (DS PSD specifications), the applicant used the DS PSD measurements as the model input. This inconsistency in model input approaches was challenged, as it demonstrated that a single dissolution/DS PSD model input could not adequately address both QoIs.

A detailed discussion followed on the challenges of DS PSD measurements. DS PSD data reflect the DS, which may differ from the DS PSD once formulated and processed into the DP. Different manufacturing processes, such as direct compression and wet granulation, can result in differences in the DS PSD in the DP. The size of the DS and morphology, whether needle, plate, or spherical, should be measured after processing, although this was recognised to be a difficult task.

Creating a link between laser diffraction-measured PSD and dissolution profiles was identified as a potential way forward. A quality regulator suggested that when making claims around DS PSD specifications and their lack of impact on dissolution, applicants should ideally manufacture DP batches with the same DS PSD (measured with laser diffraction) using different DP processes. Then a comparison of the dissolution profiles should be performed. If the profiles are similar, then claims around P-PSD and lack of effects on in vivo performance when it comes to process-related changes may be justified, provided that the dissolution method used is discriminant to that quality attribute.

The adjustment of physiological fluid volumes to fit available clinical data was also challenged. It was agreed that if certain physiological parameters, such as colon fluid volumes, are frequently modified, population files should be updated for all compounds. PBBM should ideally follow a bottom-up approach, assuming that the human physiology is known and therefore should be the same in all cases. The fluid volumes used in the models are based on observed magnetic resonance imaging (MRI) data, although different PBBM software packages use different approaches to describe fluid volumes. The mucus volume is now also considered, and the drug’s diffusion coefficient in mucus can be used as an input. Research from the University of Uppsala studied the diffusion of drug moieties through mucus (which is a complex matrix comprising 30% lipids in dry weight) and reported that lipophilic drugs diffuse more slowly in mucus, which may lead to slower access to the mucus water volume during the GI transit time compared to a more rapidly diffusing drug [38].

Stomach residence time and gastric emptying, the effect of water intake on particle transfer and the ionisation of the drug should also be considered. A publication from the University of Michigan [39] discusses the phases of gastric emptying responsible for multiple peaks, as seen in the lesinurad case study discussed earlier. Currently, no software considers partial gastric emptying, and this should be a future objective.

While significant progress has been made in how dissolution of drug products is modelled prior to PBBM introduction, disintegration is often not accounted for or bundled with dissolution. Capturing this process is very important, especially for weak acids with slow disintegration/dissolution in the stomach, since the size of the dosage form in vivo may control gastric retention until the initiation of the inter-digestive migrating myoelectric complex phase 3, in the fasted state, and may equally delay gastric emptying in the fed state.

Throughout the discussion, the group agreed on several aspects. Industry should clearly communicate its chosen approach and why it was chosen to the regulators in its submission. Any changes to a physiological parameter or any other input to facilitate the capture of clinical data should be justified. A sensitivity analysis (SA) within a physiologically appropriate range should always be conducted, and the results should be discussed by the applicant. Any limitations or caveats of the model should be made clear.

The importance of examining individual data was highlighted, rather than using mean profiles to understand the critical product attributes. Lastly, it was acknowledged that parameterization by default should differ for compounds with different physicochemical properties. For example, dissolution should be handled differently for a weak base compared to a weak acid.

The topic of conducting virtual bioequivalence (VBE) trials was briefly discussed. The importance of capturing within-subject variability (WSV) and respective confidence intervals (CIs) was acknowledged. Regulatory scientists confirmed that, in a virtual setting, simulating ten VBE trials and passing at least eight out of ten, in the context of 80–120% BE Criteria, would be a suitable acceptance criterion for VBE simulations seeking to demonstrate comparability.

#### 2.8.3. “PBBM Development and Use of VBE to Support the Use of Out-of-Specification Batches” Case Study Presented by Sumit Arora—J&J, PBBM by Nico Holmstock—J&J

This case study considered the use of PBBM in demonstrating that an out-of-specification (OOS) result obtained with a QC dissolution test could be considered not physiologically or clinically relevant for a tablet batch on stability.

Two QoIs were described for a scenario in which OOS results were obtained for a regulator-approved Quality Control (QC) dissolution test that is considered not physiologically relevant: (i) Could a recall be avoided for the OOS batch based on virtual bioequivalence (VBE) to the reference product? And (ii) how could the QC dissolution method and/or specification be modified to avoid further OOS results?

The context of use for the PBBM was to enable VBE trials to be performed, which would demonstrate that the OOS batch is bioequivalent to the reference batch and therefore eliminate the requirement for a clinical study. A PBBM was developed using a 2-step dissolution test where dissolution in simulated gastric fluid was followed by simulated intestinal fluid as an input and was adequately validated using clinical data from a non-BE batch. PBBM was then used to simulate the plasma concentration time profile of the OOS batches and to perform VBE trials between the OOS batch and the reference product batch.

The DS is a BCS class II drug (low solubility and high permeability). The commercial formulation is an immediate-release (IR) solid oral dosage form designed to be taken with food. A PBBM was developed in GastroPlus v9.5. As an input for the dissolution model, a 2-step dissolution test was applied, mimicking the physiological gastrointestinal conditions in fed-state humans. During the first dissolution phase, the tablet dissolution was measured for 60 min in phosphate buffer at pH 4.9, after which concentrated FeSSIF was added to obtain standard FeSSIF, in which dissolution was measured for another 180 min. A Z-factor was fitted to the PBDT profiles to integrate the dissolution data into the model. The distribution, metabolism, and excretion of the drug were fitted with a compartmental model using oral solution data, assuming a fraction absorbed of close to 1. The PBBM was validated by simulating the outcomes of an rBA study, which successfully distinguished between BE and non-BE drug formulations. Once the model was validated, VBE trials were conducted by comparing the OOS batches against a reference batch, which was predicted to be bioequivalent for both C_max_ and AUC_0–72_ under fed conditions. In conclusion, the overall PBBM approach was considered satisfactory, indicating that no impact on drug exposure is anticipated for the two stability batches that exhibited OOS dissolution results during QC dissolution testing.

#### 2.8.4. Breakout Group Discussion

The breakout group discussion was facilitated by Mark McAllister (Biowaived) with Mary Malamatari (MHRA) and Mariana Guimares (ESQlabs) as scribes. This case study was previously discussed in the summary reports from the 2023 M-CERSI meeting [6,17].

**Q1: Were the QoI and CoU for the PBBM sufficiently clear?**

The group considered the first question of interest to be clear; however, based on the data presented, the second question was not discussed. During the session, it was emphasised that model risk is connected to model influence, and therefore, the role the model will play, together with the totality of evidence that was used in the discussion to show that the OOS batches did not compromise product performance, including the consequence of the decision, i.e., patient safety and efficacy, is important. In this case study, model risk, model influence and decision consequence were considered to be low, medium and low, respectively [33]. The regulatory impact was considered medium/high since it would lead to waiving a BE study. The group discussed how the regulatory impact can be derived. In this case study, the risk was considered to be medium because BE and non-BE data were available to build confidence in the model’s predictions prior to model application; however, no BE data were available for the specific OOS batches. The model risk influences how strict the model evaluation and validation should be.

**Q2: Was the model sufficiently (mechanistically) descriptive and verified/validated?**

The group discussed the model build workflow and validation/verification. IV data were not available to validate the distribution and elimination components of the model. In the absence of IV data, oral solution data should ideally be used. There were experimental challenges in measuring Caco-2 permeability; therefore, the value used in the model was predicted by the ADMET predictor. When a parameter is predicted, a parameter sensitivity analysis should be used to complement the analysis report. Measured values are always preferred, but this is not always an option. Predicted and optimised permeability can be acceptable with appropriate justification and validation of the PBBM, and therefore, scientists should seek to build an appropriate, clear rationale into the report submitted to regulatory authorities.

A two-step dissolution test using biorelevant media was used to replicate the fed-state dissolution [17]. A Z-factor fitted to the two-step dissolution test profile was used to integrate the dissolution data into the model. When using the Z-factor approach with two-stage dissolution, the in vitro data for both phases of the dissolution experiment should be analysed using a pH-dependent Z-factor approach [40]. The Z-factor approach is a semi-mechanistic input for a PBBM that “lumps” parameters such as disintegration and excipient effect into the same parameter. When faced with the decision of whether dissolution data is appropriate as input for a drug product, one should consider using the totality of the available evidence, including the use of complex dissolution models as supporting information. Additionally, the details of how the dissolution tests were conducted should be discussed in the regulatory package, e.g., media composition, use of peak vessels. It was also discussed whether using a product-specific PSD would be more appropriate; however, it was agreed that both methods have limitations as both are strongly dependent on fitting a product-specific parameter to the dissolution data, meaning that both methods are dependent on the quality/appropriateness of the dissolution data.

The model validation criteria were then discussed. Although the PBBM showed a slight trend for underpredicting C_max_, it remained fit for its purpose as all predictions were within the 0.8–1.20-fold ratio. The availability of a non-BE batch was discussed, and the challenges of obtaining a non-BE batch were shared. The non-BE batch in the case study was pivotal to both the QoI and CoU and was developed to explore flexibility in manufacturing, leading to a drug product with good performance (low biopharmaceutics risk). However, the more rigorous the drug product development, the more difficult it will be to obtain a non-BE batch. PBBM is being used earlier in drug product development by innovator companies, which makes it increasingly challenging to obtain non-BE batches.

**Q3: Have you had similar questions, and if so, how did you approach them: with a similar or different approach(es)?**

Another option to address these types of question would be to change the formulation or the dissolution method; however, both options were considered less than ideal as it would have meant a restart to the development process at a very late stage. A clinical study could be used to address these questions and compare the OOS formulation with the reference. If using PBBM, the totality of the evidence could be strengthened if a link to therapeutic window and PK/PD can be established.

#### 2.8.5. “Utilization of PBBM for Defining the Dissolution Edge of Failure and Establishing Safe Space for a BCS Class I Compound (A Retrospective Analysis)” by Aishwarya Ravi, Pfizer

Fluconazole was selected, as it provides an opportunity to show that PBBM approaches can provide additional bioperformance insights for BCS 1 compounds. The model was developed primarily to (1) showcase that PBBM can be used to support biowaivers of BE studies where dissolution f2 similarity testing may fail, and (2) propose a potentially wider dissolution safe space range that would expand beyond the current dissolution ranges provided solely through BCS classification.

The model was developed utilising data from prior pharmacokinetics (PK) studies, including formulations such as an intravenous infusion, an oral solution (in a single ascending dose study (SAD)), oral capsules (in rBA/BE, food effect, and PPI studies), and an oral tablet (in a rBA study). The model was developed in GastroPlus, starting with IV PK data to establish the clearance and volume of distribution parameters for fluconazole. The model was then further expanded to include absorption from oral dosage forms (solution, capsules, and tablets). Dissolution of fluconazole was modelled in two ways: (1) utilising the Hintz–Johnson model when in vitro dissolution data were not available, and (2) a Weibull dissolution model to describe in vitro dissolution profiles. For certain dissolution datasets, where the reported experimental data did not achieve complete dissolution by the final timepoint, the dissolution profile was extrapolated with a Weibull function to generate a complete dissolution profile. The developed PBBM was verified to adequately predict the absorption phase of fluconazole PK across a range of formulation types and under different prandial states (fasted, fed, PPI). The performance of the model was evaluated against a predicted vs. observed (P/O) acceptance range of 0.80–1.20 for C_max_ and AUC.

Model predictions pertaining to oral solution PK studies showed high AUC P/O ranging from 1.18 to 1.80 across doses. These high P/O values could be attributed to lower values of observed terminal half-life in these studies relative to the terminal half-life from IV data as well as a smaller number of subjects in the oral studies relative to the IV study; therefore, some questions remain regarding the model’s ability to predict the PK. Model predictions for the fasted state incorporate data from the fasted arm of a food-effect study, which suggested a high P/O ratio for AUC (1.40). However, the absolute mean AUC value fell well within the in vivo variability range observed for AUC values for that study. Nonetheless, the predicted P/O for C_max_ values were within the 0.8–1.2 range across doses and dosage forms, except for one solution dose, which showed a value of 1.21 in 18 out of 19 simulations.

The verified model was then used to conduct VBE trials. The model was able to demonstrate BE between two capsule formulations that exhibited f2 < 50, replicating the outcome of a BE Study. This BE study aimed to compare formulations that underwent changes in manufacturing and production sites. The model was additionally verified by evaluating BE between a tablet and a capsule formulation, as well as for two capsule formulations dosed as a single unit (150 mg) vs. multiple units of a lower strength (3 × 50 mg). Additionally, VBE was utilised to identify the lower limit of the dissolution safe space for fluconazole capsules. These VBE results suggest that the dissolution safe space for fluconazole capsules could be extended to Q = 80% at 75 min, with a 0.1 M HCL 100 rpm basket as the dissolution method, which is significantly longer than the typical limit of Q = 80% at 30 min (or faster) for rapidly dissolving BCS 1 compounds that is required for biowaiver applications. These outcomes demonstrate the potential advantage of including PBBM as part of BCS 1 compound drug product development to achieve a biowaiver and generate a dissolution safe space.

#### 2.8.6. Breakout Group Discussion of Case Study 9

The breakout group discussion was facilitated by Nikoletta Fotaki (Bath University) with Nena Mistry (GSK) and Laurence Dodd (ESQlabs) as scribes.

**Q1: Were the QoI and CoU sufficiently clear?**

The group considered the QoI and CoU to be sufficiently clear; however, there was some discomfort given the f2 dissolution similarity failure (i.e., f2 < 50). The discussion went on to the failed f2 batch that exhibited a slower dissolution profile despite having passed the specification set to ensure product quality, leading to debate about balancing product quality and clinical relevance for BCS1 molecules. It was widely agreed that if there was uncertainty in the data input to the PBBM, the uncertainty would be propagated throughout the model.

**Q2: Was the model sufficiently (mechanistically) descriptive and verified/validated?**

The group discussed a range of topics, including the choice of drug, which was considered optimal as it demonstrated linear PK with no gut metabolism. From a regulatory perspective, a substantial amount of data was provided, consisting of historical information on the compound that was used in the development and validation of the model. However, it was not fully accessible for independent review at the time. From a traditional BE study point of view, a P/O outside of 0.8–1.2 raises some concerns. Dissolution was limited at 30 min; however, the group expressed confidence in allowing the extrapolation of the profile using the Weibull dissolution model to predict the complete profile; beyond the specification, the final stages of dissolution are known to contribute minimally to absorption for a BCS Class 1 compound. The Johnson model was used for oral solution data where no dissolution was present. Weibull was used for tablet and capsule data where the dissolution was slow. The Weibull dissolution model was utilized to extrapolate tablet and capsule dissolution profiles to 100% drug release. A common limitation associated with the use of the Weibull model for disintegration and dissolution modeling was discussed. A more general question was then debated on why a PBBM would be required when the slower dissolution was eligible for a BCS 1 biowaiver. The regulatory perspective requires that, if a formulation undergoes certain types of change, then either a BE must be completed, or justification should be submitted. If the formulation had not been changed, then indeed this exercise would likely not have been completed. For PSD data as a model input, the largest d50 was used as the input and worst case, in lieu of actual data. The observed intrasubject coefficient of variation (ISCV) for both Cmax and AUC from the BE study was used in the VBE simulations to capture the variability observed in the actual clinical data.

The model PK parameter prediction was discussed and showed a consistent overprediction for AUC in the SAD study. The group noted that insufficient clinical data were available for certain doses across different dosage forms to fully justify the clinically observed AUC range. Additionally, the doses with AUC overprediction had limited subject numbers, leading to a narrower observed range, which hindered the model’s ability to capture the full variability. Given the small sample sizes, only single simulations were possible, as population simulations could not be performed due to insufficient data being available to reflect the clinically observed variability.

With respect to model validation for the context of use, a non-BE batch would help to demonstrate that the model is predictive, as only a change in T_max_ was observed in the food effect study. All other changes, such as DS particle size, do not affect the PK profile, therefore leaving the model’s reliability in predicting failure in question. The consensus was that a parameter sensitivity analysis would be beneficial, particularly focusing on formulation properties and physiological variability.

**Q3: Have you had similar questions, and if so, how did you approach them: with a similar or different approach(es)?**

The application of Weibull to capture dissolution is a relevant method for BCS1 APIs, as it has no solubility or volume limitations; however, this may not extend to other BCS class compounds, as it is not a biopredictive method. In this case study, full release was not achieved within the defined time points; therefore, using Weibull to extrapolate release to the full extent was acceptable. To develop a more mechanistic PBBM, it was appreciated that using Weibull for release modelling rather than dissolution modelling is an appropriate and key differentiation. **Additional comments:** During the breakout session, the regulator who reviewed this case study for the M-CERSI 2023 workshop [6,18] shared valuable insights regarding the regulatory process and model considerations. They acknowledged that had there been an opportunity for the company to engage directly with the reviewer, timely replies, feedback, and addressing specific model-related comments raised during the review could have been provided. This interaction would have enabled discussion and resolution of questions surrounding the model’s validity for requesting a biowaiver through PBBM. As this case study was conducted within an academic framework, the usual rigorous communication between the company and the EMA was absent. This limited interaction may have constrained the company’s ability to fully explore the model’s capabilities and scope with regulators. The regulator’s feedback provided clarity on the regulatory context and highlighted the importance of proactive, structured dialogue in future cases. In conclusion, for this case study, it is believed that the presented PBBM would not be accepted as a substitute for, or to waive, a BE trial to support a marketing authorization. However, the concerns indicated above could be resolved during the application procedure.

## 3. Day 2

### 3.1. Summary of Presentation: “Lessons Learnt, Gaps and Future Horizons on PBBM from a Regulatory Affairs Perspective” by Mary Malamatari, MHRA and Øyvind Holte, Norwegian Medical Products Agency (NOMA)

The PBBM concept is relatively new, at least in terms of supporting new and existing marketing authorisations. To date, the European and UK regulatory agencies have had limited experience with case handling of PBBMs. They have, however, had extensive experience with related concepts, i.e., modelling in relation to PBPK and quality.

In many ways, the current situation for PBBM resembles the introduction of “the new quality paradigm” 15+ years ago. Quality by Design (QbD) represented a new way of demonstrating the quality of a batch, and in addition, provided powerful tools to support the development and lifecycle management of a drug product and the manufacturing process. The introduction of QbD concepts in regulatory submissions was not easy at the time, for several reasons. There is a risk that PBBM will face some of the same hurdles as QbD did, and therefore, it is useful to learn from the QbD story.

In his presentation, Øyvind Holte provided a few examples of typical model approaches from the QbD area. Although the purpose and application of these models are different than PBBM, both QbD models and PBBM are used to predict critical properties of drug products that would otherwise have to be determined by traditional analysis.

Both QbD models and PBBM can be classified according to risk, impact, and criticality. From a regulatory perspective, the level of documentation and scrutiny of assessment should be aligned with the impact on the regulatory decision. For example, will the model replace an otherwise necessary set of data/observations (high impact), or is the model used primarily to guide the development of the drug product, which is later shown by traditional means to be adequate (low impact)?

Despite the regulatory guidance established to support QbD, it appears that the full potential of its concepts has still not been achieved. It is acknowledged that many companies use the QbD concepts in-house during pharmaceutical development to arrive at a robust formulation and manufacturing process. However, the regulatory flexibility and efficiency represented by the design space and real-time release testing are applied to a lesser extent.

A successful implementation of PBBM as a widespread tool in regulatory applications could be supported by unmistakable terminology, agreed-upon approaches for development and validation, and suitable training.

Mary Malamatari presented on her regulatory experience within the UK, which is primarily with PBPK models in applications supporting drug interactions. However, PBBM would resemble PBPK in many ways in terms of the verification, validation and qualification of models.

The regulatory assessment of new drugs needs a cross-functional assessor team of pharmaceutical, non-clinical, medical, statistical, clinical pharmacology, and compliance assessors. These groups would work collaboratively on the assessment of the application.

When developing models, it is important to apply available guidance and learnings from previous initiatives, e.g., IMI OrBiTo, when considering what is required to support the models. This project demonstrated that consistent use according to the standard operating procedures (SOPs) of software is essential to achieve reliable results. SOPs are also essential in PBPK/PBBM applications, to move them from being an exploratory tool for developers to a regulatory decision tool [41].

A number of applications for PBBM are expected in regulatory submissions: formulation design and development at earlier clinical stages, clinically relevant product specifications (e.g., dissolution or particle size distribution), biopharmaceutics risk assessment and optimal design for clinical studies (e.g., food effect, pH-mediated drug interactions with acid-reducing agents), and bridging formulations during development or post-approval by waiving clinical studies through VBE testing and establishment of a safe space for QA/QC and post-approval changes (e.g., allowing OOS, widening specifications or manufacturing site changes).

Maria Malamatari presented the key messages derived from the roundtable discussions by Regulatory Agencies at the 2023 M-CERSI meeting. The following main deficiencies within PBBM submissions were discussed [6]:

- Unclear model objectives.
- Inadequate model development (e.g., input parameters and/or dissolution input methods not sufficiently justified).
- Uncertainty in parameter estimates.
- Inappropriate model validation.
- Not sufficiently justified pre-specified acceptance criteria.
- Drug product-specific anomalies.
- Incomplete understanding of product CMAs, CPPs, and CQA relationships.
- Uncertainties on drug disposition due to lack of IV data.
- Uncertainties on within- and between-subject variability on simulations.

In her closing comments, the presenter reflected on the future steps for promoting the use of PBBM as a regulatory decision tool. Collaboration in workshops such as this, which encourage interaction between industry, regulatory agencies, and academia, can be of great significance in evolving the use of PBBM throughout the drug product development, regulatory approval and lifecycle management of a medicinal product.

### 3.2. Summary of Presentation: “PBBM Template—A Cross-Industry and Regulators Collaboration” by Sumit Arora, J&J

The progress made on the PBBM report template, developed through collaborative efforts between industry and regulatory stakeholders to streamline submissions from industry and facilitate health authorities’ reviews, was presented. The idea of the PBBM template originated from the 2023 M-CERSI PBBM workshop, where there was a clear indication from industry and regulatory colleagues that a PBBM template would be helpful to summarise the base requirements for model development, verification and validation, and how they should be presented to minimise PBBM rejections [6].

The presentation discussed the overview of the PBBM report template, which comprises eight main sub-sections and is described in detail in Arora et al. [11].

1. Executive Summary;
2. Question of Interest and Context of Use;
3. Assessment of Model Risk and Regulatory Impact;
4. Background Information;
5. Materials and Methods—discusses modelling strategy, model development, parameterization, and validation criteria;
6. Results—Presents model validation outcomes, performance results, sensitivity analysis, and application results;
7. Discussion—Offers insights into model prediction performance and acknowledges its limitations;
8. Conclusions.

The PBBM report template is sufficiently descriptive and aims at providing suggestions on how the model could be parameterized and validated; however, it is not an all-inclusive template. Depending upon the QoI and CoU of the PBBM, different sections can be added or removed from the template. The authors clearly highlighted that this effort was by no means a regulatory guideline, but instead summarised the current thinking around the best practices and considerations for PBBM submissions.

In addition, the presentation highlighted various themes/topics that were identified during the combined review of the report template by the industry and regulatory participants. A detailed discussion on the different themes can be found in Arora et al. [11].

Some of the topics which were discussed during the presentation were:

A. **Need for aligned terminology**—Currently, there is a lack of aligned terminology for model evaluation and acceptance, which hinders effective communication. The PBBM report template manuscript aimed to provide a glossary of key terms used in the PBBM report template.
B. **(QoI), (CoU), model risk assessment and regulatory impact**—Tangible examples of how the above could be framed for various PBBM applications. For more details, please refer to the published manuscript.
C. **Considerations for drug product variants and fit-for-purpose clinical pharmacokinetics data for PBBM**—This theme provided insights on what kind of drug product variants sponsors should leverage to validate their models. This theme also discussed what alternate clinical data sponsors could leverage in the absence of a non-bioequivalent batch, as is ideally required for model validation.
D. **Model development and parameterization**—This is the most detailed section in the PBBM report template, with suggestions to sponsors on how to parameterize a PBBM. In addition, during Day 1 of the 2023 PBBM workshop, presentations and breakout session discussions proposed best practices for measuring solubility, dissolution, supersaturation, precipitation, permeability, and modelling dissolution profiles [18].
E. **Model validation and acceptance criteria**—This theme discussed several performance indicators and their acceptance criteria. However, the acceptability of certain criteria should be evaluated considering the QoI and CoU of the PBBM, model risk assessment and model impact. The higher the model risk and impact, the more stringent the acceptance criteria should be.
F. **Model application**—VBEs are usually conducted at the model application stage to answer the QoI. This theme presented considerations needed to design VBE studies, and highlighted gaps such as the lack of adequate characterisation of inter- and intra-subject variability of gastrointestinal physiological parameters, which requires additional research.
G. **Model reuse**—It is not uncommon for a PBBM to be used for multiple drug product questions. This theme discussed how model reuse should be approached and briefly mentioned the concept of MMF proposed by the FDA, in which model sharing is one of the cornerstones.

### 3.3. Summary of Presentation: “The Use of Simple Oral Absorption Models (BCS/DCS) and Biorelevant Dissolution Methods in Biopharmaceutics Risk Assessment” by James Butler, GSK

Regulatory guidance on biopharmaceutics risk assessment is, by necessity, conservative, considering what could happen in a worst-case scenario. In the context of safety and efficacy for patients, an ideal risk assessment for regulatory purposes, therefore, has some bias, as it has minimal tolerance for false negatives.

**Figure 5:**
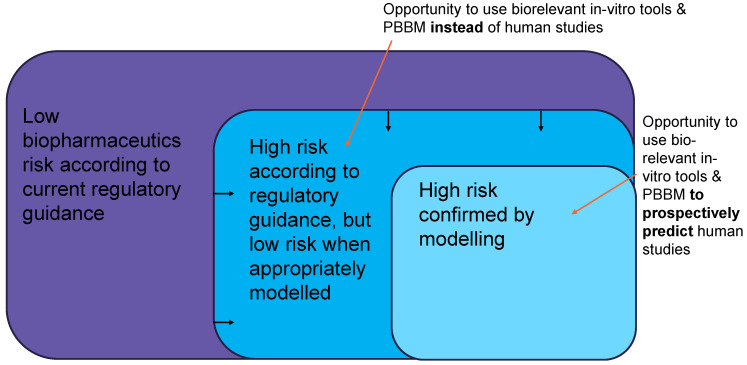
Opportunities to narrow the gap between regulatory risk assessment tools and tools used in early development risk assessment.

In early development, by contrast, biopharmaceutics risk assessment is less conservative, as the questions asked are different. Typically, the question is “*what is most likely to happen?*” For example, in predicting the degree of the developability challenge, over- and under-prediction are equally undesirable. Therefore, the ideal risk assessment is often equally balanced between over- and under-prediction. By understanding the different drivers for biopharmaceutics risk assessments, not only can a mutually beneficial understanding of biopharmaceutics risk assessment be gained, but there are opportunities to repurpose tools used in early-stage risk to address regulatory considerations, such as biowaivers (Figure 5).

There is a trend towards the application of tools that are more frequently used in early development to address regulatory questions. For instance, DCS/rDCS was originally developed for oral developability assessment [26]; however, recent publications have identified its potential to be extended into biowaiver justification [42,43]. The use of biorelevant media in solubility and dissolution assessments, rather than the simple aqueous buffers recommended by BCS, may also be a way to close the gap between considering the “worst case” and “most likely case” scenarios without adding undue risk for patients.

PBBM has been specifically referenced in recent regulatory guidance, such as the recently published FDA guidance on pH-dependent drug interactions with gastric acid-reducing agents [44].

Therefore, biopharmaceutics tools that are more typically used in early development, such as rDCS, PBBM/PBPK and biorelevant dissolution, have emerging potential to inform better regulatory decision making.

### 3.4. Summary of Presentation: “General Principles for Describing Formulation Performance Using Dissolution as an Input to PBBM” by Mark McAllister, Biowaived

This presentation on the general principles for describing formulation performance as an input for PBBM began with an overview of the regulatory positioning of the need to fully integrate biopredictive dissolution testing with PBPK/PBBM to use modelling to inform oral drug product development. A typical biopharmaceutics toolkit, comprising in vitro and in silico tools, was reviewed with a particular emphasis on the challenges posed by formulation complexity, API solubility and the physiological biorelevance of test methods for developing a biopredictive dissolution method. Several physiological processes, such as motility and transit, fluid composition variability and segmented luminal distribution, epithelial permeability, and dynamic digestion methods, are not typically reproduced by dissolution methods. Despite numerous attempts to develop technologies to overcome these limitations, routine biorelevant methodologies which can holistically address these challenges remain elusive. The type of dissolution data also changes as a project transitions from early preclinical development to clinical and commercial development stages, necessitating thoughtful consideration of how this change can be integrated within a PBBM, which is also under continuous evolution and development across the product lifecycle. Options for inputting dissolution data to PBBM spanning a range of complexity were compared in terms of their sensitivity to physiological regional differences (and associated between- and within-subject variability). Simpler empirical models, such as a direct data inputting or the Weibull function, were less sensitive than semi-mechanistic dissolution models, such as lumped parameters/models, e.g., the Diffusion-Layer Model (DLM) or Z-factor, which in turn were less sensitive than the more or fully mechanistic dissolution models such as the Noyes–Whitney and extended Wang–Flanagan models. When the same series of input options was assessed for their applicability to represent the performance of complex formulations, the order was reversed, with the simpler empirical models judged to be the most readily applicable approach. In general, it was suggested that there is a need to understand the limitations (and their impact on how other gastrointestinal (GI) parameters (e.g., in vivo solubility) are handled in simulations) associated with the different options that exist for describing dissolution performance within the different software platforms that are typically used for PBBM. Mechanistically understanding the different physicochemical factors that drive dissolution (solubility, solubility product, bile salt partitioning, supersaturation and precipitation) through well-designed lab experiments can help with model parameterisation. There is also an opportunity to develop datasets that go beyond the average and describe behaviour across relevant ranges of physiological variability. Ultimately, the impacts of formulation composition and drug product processing methodology are still not fully described in typical PBBM dissolution models (e.g., wettability, lubricant effects, porosity, DS PSD change during processing), and this necessitates taking a more considered approach to accurately describe dissolution performance. Whilst complex GI simulators can be used to profile product performance in a way that addresses some of the aforementioned physiological limitations of conventional systems, it is also true that the integration of data from these systems can require re-modelling of the data to address specific system-related parameters that contribute to the dissolution profile obtained. Biopredictive dissolution testing of bioenhanced formulations, which are designed to address limiting molecular biopharmaceutics properties for absorption, requires careful consideration. In some cases, formulation performance can be the direct impact of GI processing/digestion, e.g., lipid-based formulations, and for amorphous solid dispersions, complex solution speciation can occur and needs to be accounted for when building a mechanistic absorption model to facilitate the use of dissolution data in PBBM. In conclusion, it was suggested that there are opportunities to innovate with PBBM and “*Go beyond the average*”, with potential for different patient populations and disease states to be more comprehensively served.

### 3.5. Summary of Presentation: “Clinically Relevant Dissolution Specifications, Implication for Use in the Post-Approval Setting and Tension with f2 Similarity Testing” by Paul Dickinson, SEDA

This talk focused on drawing out the facts and emotions surrounding the use of CRDS in a post-approval setting and posed two questions:

- What is the value of CRDS in the post-approval setting?
- What are the tensions and challenges?

The talk focused on dissolution; however, the concepts were likely valid for dissolution inputs for PBBM models (or IVIVC), and they introduced the following key concepts:

- BCS1/3 like product risk;
- Product Specific SUPAC/”MyPAC”.

It was noted that CoU and the totality of the data would be important in any specific discussion.

For the speaker, the value of CRDS pre-approval was clear, in that a specification is set on clinical performance (patient benefit), and for the sponsor (and regulator), it can reduce the discussion about the dissolution test conditions and lead to early agreement on dissolution conditions and specifications, which substantially derisks the drug product dossier.

The talk limited the scope of CRDS to BCS2 and BCS4 compounds in IR products; however, it noted that for dissolution methods where limited/no in vitro discrimination is expected (e.g., BCS1 and BCS3), the general expectation was to apply f2 testing to prove similarity for a “change” (e.g., SUPAC-IR and ICH M9 with no f2 if very rapid dissolution).

After introducing the concept of safe space, a question was raised of whether f2 testing is appropriate in the case of CRDS when the safe space has been “proven” or even when IVIVC/R has been established, if the specification has been set to discriminate between acceptable and unacceptable batches.

Importantly, it was noted that lower risk BCS2 and 4 compounds and well-designed drug products may have rapid dissolution compared to physiological processes, and so a safe space is a likely outcome, and thus limited in vitro dissolution discrimination is expected [45]. Therefore, it could be argued that the regulatory lens through which the review of the compound in that particular product is performed changes from a high biopharmaceutics risk to a biopharmaceutics risk considered to be “BCS1/3 like”. The onus would be on the sponsor to show that the biopharmaceutics risk is a “BCS1/3 like risk”, which would likely focus on providing dissolution and clinical performance data similar to that expected for a BCS 1/3 compound/drug product. Furthermore, it was assumed that as CRDS is limited to BCS2 and BCS4 compounds in IR products, there would be a requirement for a product-specific Quality Risk Assessment (QRA), which would lead to the identification of the highest risks to in vivo dissolution (failure modes and effects analysis, FMEA) and the subsequent generation and testing of variants (side batches) with the potential failure modes identified as highest risk as part of development. This is analogous to the regulatory risk assessment generally applicable to any drug product and described in guidance such as ICH M9, SUPAC-IR and SUPAC-MR, which are based on scientific and societal experience/data.

The speaker then imagined two “extreme” approaches to the application of CRDS for regulatory flexibility in a post approval setting:

1. All changes could be supported by the dissolution method without a BE study, versus only Level 2 SUPAC changes being supported by the method;
2. Changes could be supported by passing specification, versus similarity via f2 testing needing to be proven.

Statement Pair 1 was interrogated in detail as shown in Table 1.

The speaker noted that this is project-specific, so we cannot decide which of the factors discussed in Table 1 would be most important, and reiterated that SUPAC-IR is a risk assessment based on the state of the knowledge at the time and generically covers all products. However, for CRDS there is a product-specific dissolution method built on highly specific data for that product (informed by QRA, FMEA and experimental work), and consequently a potential solution for the application of CRDS post-approval is:

- To define, as part of the original approval, the type of changes where the dissolution method and specification is still likely to be relevant.

This could be in the form of a QRA. For example, if API particle size was constrained in the initial submission and not investigated in the CRDS work up, could any API characteristics changes be supported? This would be analogous to concepts in the FDA IVIVC guidance and could be mutually agreed upon during review (“Product Specific SUPAC”/”MyPAC”).

The second pair was discussed in less detail; however, it noted that challenges may be focused on pharmaceutical control/quality of the manufacturing process. These may be better addressed through continual process verification, and it was highlighted that robustness of supply is an important aspect of pharmaceutical quality if there is no impact on the patient/PK.

### 3.6. Summary of Presentation: “The Development and Framework of MMF as a Regulatory Initiative” Presented by Lanyan (Lucy) Fang, FDA and Reported by Claire Mackie

This talk shared the development of the FDA Model Master File (MMF) initiative from the Office of Generic drugs (OGD). The goal of the ODG is to facilitate generic drug development and review along with policy development and regulatory decisions. Modelling and Simulation (M&S) is a modern tool for drug development, and MIDD is an integral part of all new molecular entities (NME), new drug applications (NDAs) and Biologics Licence Applications (BLAs). Model-Integrated Evidence (MIE) can cover many challenging areas for generic drug development, especially for complex generics. It is also clear that new modelling types and utilities such as artificial intelligence (AI) and machine learning (ML) are becoming relevant. M&S can be used for questions in both the oral and non-oral drug product delivery areas, such as ocular, nasal, pulmonary (orally inhaled), dermal, and gastrointestinal, to predict both local and systemic concentrations. Modelling approaches in these areas include PBPK, computational fluid dynamics (CFD), and semi-empirical and quantitative systems pharmacology (QSP). Two drug product areas were highlighted using examples from regulatory decision-making in the OGD, including oral drug products, such as (1) risk assessment of the impact of DS PSD on BE, (2) risk assessment of deviating dissolution profiles on BE, (3) biowaiver assessment, and in the non-oral space, a locally acting dermal product. The key message from all examples was the criticality associated with the verification and validation (V&V) of the model.

The FDA has been encouraging the use of quantitative methods and M&S with MIE to support the development and approval of generic drug products, and this has been increasing steadily since 2018. The MIE pilot programme was launched in October 2023 as a dedicated regulatory platform to foster early and focused interactions between industry and the FDA on MIE approaches for establishing bioequivalence (BE) in generic drug development. The aim here was to benefit both industry (time, cost, alignment and clarity) and the agency (efficiency, reduce number of drug approval cycles, and develop an ecosystem with industry to develop effective BE approaches). Learnings from this program, together with earlier workshops in both 2021 and 2022, included clear benefits in model sharing, standardisation, and archiving in regulatory submissions. In May 2024, a workshop was convened to discuss the considerations and potential regulatory applications for an MMF. MMFs are information and data in a quantitative model or modelling platform that have undergone sufficient V&V to be viewed as portable, reusable, generalizable, and sharable from an FDA regulatory perspective. The advantages include reuse, scalability, consistency, and the support of an ecosystem. They are also intended to accommodate the dynamic nature of models and data, permitting multiple models to be applied for the same use and encouraging scientific publications, building venues and platforms for public access to non-proprietary knowledge/information. An MMF should be submitted as a subtype of the drug master file (DMF). An MMF submission should include the regulatory QoI and CoU, scientific rationale, modelling analysis plan and report, model files, datasets, literature, and all sources of information that have been developed and utilised to support the development of the MMF. Potential types of MMFs include (a) a product- or an API-specific model, (b) a methodology or a good modelling practice that can be applied to multiple products for the same purpose of use, (c) a verified and validated virtual physiological organ or system that can be subsequently connected with drug product-specific information for the established use.

### 3.7. Summary of Presentation: “Options to Progress a Proposal for a Harmonized PBBM Guideline” by Claire Mackie, J&J & Matt Popkin, GSK

The topic of PBBM or PBPK in biopharmaceutics has been gaining momentum since 2017 through a series of meetings and workshops involving industry and regulators in both the US and Europe, including the topics of translational modelling in chemistry, manufacturing, and control management (CMC) space and CRDS. The most recent meeting in August 2023 set up a scientific collaboration where representatives of industry and six global health authorities (FDA, EMA, MHRA, ANVISA, Health Canada and PMDA) came together with the main goal of debating the views of the scientific community and to establish best scientific practices in building PBBMs [1,6,17,18]. The final write-up was an industry-led template and manuscript detailing the key scientific elements to be included in a PBBM report when submitting to health authorities, which has since been published [11].

The idea for an ICH guideline on PBBM has been growing through the series of meetings noted above and, in effect, could “connect the dots” in pharmaceutical development and quality applications, between drug product quality, QbD principles, dissolution sciences and clinically relevant specifications. As PBBM is a powerful tool that can lead to enhanced drug product understanding during development, this elevates drug product quality by providing a more accurate and holistic understanding of the impact of CMC changes on the in vivo drug product performance. These efforts will contribute towards patient-centric drug product development and may allow manufacturing and regulatory flexibility throughout the drug lifecycle, which would be beneficial to industry, regulators and patients. The use of modelling to support the development of products and their control strategies is an established ICH concept, although currently, only the FDA has drafted guidance [22]; however, it is understood that other agencies have an interest in developing their own guidance. This would be a great opportunity for industry and regulators to lead the way for cross-agency harmonisation.

With this in mind, pharmaceutical research and manufacturers of America (PhRMA) has developed a proposal for a new ICH multidisciplinary guideline for PBBM. The new ICH topic proposal is entitled “Physiologically Based Biopharmaceutics Modelling (PBBM): For Drug Product Development, Post Approval and other Quality Applications” and contains clear aims, technical and scientific issues to be addressed, objectives and expected deliverable(s). The proposal is complementary to ICH M15 MIDD, which has reached Step 2 for public comment.

The ICH M15 guidance covers general technical and regulatory principles for modelling and simulation. The proposed PBBM ICH guideline would specifically provide details around the different applications of PBBM and its technical requirements, as the context of use differs significantly and necessitates a robust background in drug product quality, QbD principles, physical chemistry and dissolution sciences. In a related manner, a complementary new ICH topic proposal for dissolution has also been developed jointly by PhRMA and the European Federation of Pharmaceutical Industry Associations (EFPIA), which considers the clinical relevance and biopharmaceutical properties of drug product dissolution. The Dissolution Quality Guideline would certainly build on and evolve from the ongoing ICH Q6 update and be complementary to the proposed PBBM guideline and M15. Collectively, the ICH guidelines should work together to enable the use of PBBM in establishing BE and supporting CRDS. Feedback on the new topic proposals was provided in May 2025 at the ICH Assembly meeting.

### 3.8. Summary of Presentation: “Introduction to Safe Space” by Xavier Pepin, Simulations Plus

**Figure 6:**
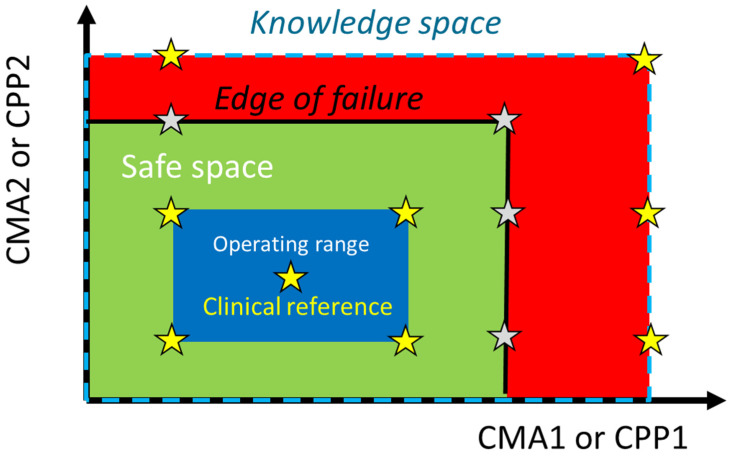
Illustration of safe space, knowledge space and edge of failure with regard to the operating range for the manufacturing of drug products. Yellow stars are batches tested in the clinic. Grey stars symbolise virtual batches tested through PBBM.

A safe space is defined by the range of quality attributes (QA) for a drug product where all the batches manufactured are anticipated to be BE to one another. Typically, safe spaces can be defined using clinical exploration only or a combination of clinical data and a validated PBBM. The idea is, from observed CQAs for the DS and DP, to manufacture clinical variants of the commercial formulation with the largest possible variations in these CQAs and test extreme variants in a clinical trial. The results of this evaluation will define the knowledge space (Figure 6). If the knowledge space is large enough, batches tested at the extreme of this space may be non-BE to the clinical reference around which typical commercial manufacturing will occur (normal operating range). Using a validated PBBM, virtual batches can be defined to interpolate batch performance throughout the knowledge space and define the edge of failure, i.e., the largest size of the safe space, where real or virtual batches will be BE to one another.

Safe spaces can also rely on the demonstration of equivalent efficacy and safety, should the safe space based on BE be too small to allow for the manufacturing of the drug product.

Many DS and DP attributes can be bound by a safe space, as long as the variation in these attributes induces a change in dissolution using a discriminant and biopredictive method. These attributes comprise but are not limited to variations in dissolution or disintegration, DS particle size or polymorphic impurity, tablet hardness, porosity or coating thickness. The development and validation of a PBBM relies on being able to link DP dissolution to PK profiles. Specifically, the DP dissolution method and discriminatory potential to variations in DP CQAs should be investigated prior to selecting the dissolution method that will be used to test clinical DP batches for integration into PBBMs. In parallel, a batch history linking clinical DS and DP to the selected clinical data for model validation should be generated. This batch history should comprise material attributes, process parameters and dissolution data for the selected dissolution methods. If the PBBM can predict observed human PK data using the dissolution of relevant DP batches as an input, then the dissolution method is called “biopredictive”. Biopredictive methods can either be QC methods or biorelevant methods, the latter aiming to mimic the volume and composition (and their dynamic changes) of the site of drug administration. The clinical data selected for model validation should also be carefully selected as “fit for purpose”, i.e., testing drug product batches which are representative of the commercial DP, and having introduced meaningful variations in composition or the manufacturing process that affect the dissolution profile of the DP. These variations should also be large enough to affect the drug PK profile.

The choice of a dissolution method and how dissolution data will be integrated in the model are therefore fundamental elements of PBBM development and will depend on model application, i.e., on the QoI. Several case studies were then presented to illustrate the concept of safe space. For zolpidem hemitartrate modified-release formulations, the PBBM was used to demonstrate that the QC dissolution method was biopredictive using a Weibull function to integrate DP dissolution [46]. For immediate release formulations of zolpidem, a safe space could be defined between a test and reference formulation, using P-PSD to integrate dissolution and by running a series of 10 VBE studies of adequate size, as in any clinical trial, i.e., considering the study design, estimated geometric mean ratios (GMR) and within-subject variability (WSV). A second case study on isoniazid illustrated a safe space between IR dissolution products using a Z-factor to integrate dissolution and factoring in the luminal drug degradation in a fixed dose combination with rifampicin [47]. A third case study on the acalabrutinib maleate tablet showed a slight advantage of the P-PSD over the Z-factor to model the in vitro dissolution of clinical variant batches and illustrated the application of a PBBM and PK-PD model to define a safe space for DP dissolution [48]. The final case study on omaveloxolone showed that a well-defined PBBM can capture complex interactions between in vivo dissolution, site of drug release and first-pass gut extraction as induced by a change in prandial state. For this case study, since the DP is the pure amorphous spray-dried DS, the in vivo dissolution was calculated based on the DS particle size distribution of the DP batch [49]. Administering DP in fed states or with acid-reducing agents could be an alternative to generating non-BE batches, if the change induced by co-administration can validate the in vivo dissolution model’s accuracy.

All the case studies presented illustrate the use and value of safe space definition to allow the setting of clinically relevant DS and DP specifications and manufacturing flexibility pre- or post-approval, following expected or unexpected changes in product quality.

## 4. Group Discussion with Facilitated Questions—Feedback from Each of the Three Breakouts

Session facilitators and scribes: Christer Tannergren, Oyvind Holte, Andrew Butler, Nena Mistry, Orla NiOgain, Mark McAllister, Nico Holmstock, Sue Cole, Claire Mackie.

Day 2 breakout sessions focused on the theme of opportunities and challenges associated with developing a dissolution safe space using PBBMs. The aim was to generate discussion on “hot topics” related to the dissolution safe space. The three identified topic areas were (1) biopredictivity of the input dissolution method, (2) defining the dissolution safe space’s upper and lower bound criteria and (3) opportunities and challenges for developing a dissolution safe space for a BCS II/IV drug using PBBM.

Breakout session 1, which addressed the topic of biopredictive dissolution, debated “what success looks like for biopredictive methods”. Overall, there was wide agreement that a match with clinical outcomes was a key indicator of success, irrespective of the input methods selected, e.g., QC or biopredictive. A decision tree to guide dissolution method choices was established at the 2023 M-CERSI PBBM workshop, emphasising distinct methods for bio-predictive and QC purposes [18]. Dissolution methods that demonstrate rank order for in vivo performance are useful for early product/formulation development, even when biopredictive criteria are not yet established. Furthermore, discussions focused on which datasets models should be able to reproduce. The need to estimate historical clinical PK data for all formulation types or just those relevant to the question and formulation of interest was debated; a recapitulation of all historical formulation performance, whilst optimal, was not considered necessary in all cases. The necessity of anticipating non-BE batches was considered highly useful in supporting model robustness. However, in the absence of non-BE data, model validation with acid-reducing agents (ARA) or food-effect data as strategies to enhance model robustness could be considered, with specific attention to how model input data and model development are performed to increase confidence. Regulatory views may differ, suggesting the need for further dialogue.

Regarding dissolution input methods for PBBM, the group discussed a preference for mechanistic input methods over direct methods, as mechanistic approaches facilitate VBEs, data extrapolation and more comprehensive calculations/estimations. Experiences highlighted some regulatory agencies favouring direct input methods, potentially due to training gaps. Challenges in understanding the effect of food on dissolution were noted, with fasted conditions typically more readily estimated than fed states. Insights from paired in vitro dissolution and FiH data from the same batch could improve understanding, supported by advanced gastrointestinal in vitro simulation tools, e.g., the Dynamic Gastric Model (DGM) and TIM-1. These tools, although not a replacement for clinical performance, can enhance model parameter understanding and selection, leading to improved prediction performance when integrated with PBBM.

Breakout session 2 was focused on defining the dissolution safe’s space upper and lower bound criteria. Initially, the group conferred on dissolution safe space terminology and whether participants were aligned on how to generate a dissolution safe space. Participants shared experiences and agreement, where formulation variants to achieve both slow and fast release profiles were most useful to confidently define the safe space, emphasising the difficulty of defining safe space boundaries using a single formulation variant. The utility of non-precipitating solutions in early clinical PK studies to assess absorption rate limitations and mechanisms was considered valuable. The difficulty of justifying a safe space using different formulation types, e.g., capsules and tablets, due to differing release mechanisms was noted, suggesting that a particle size distribution approach could help bridge this gap. Further discussion explored the potential for dissolution specifications to reflect safe space definition, including possibly using a two-point specification if a boundary was linked to a safety or efficacy concern.

Challenges in defining safe space were shared, focusing on the type and quality of data, covering several topics. Limitations of study sample sizes were noted for validating models and accounting for absorption changes due to disease states, given the predominance of relative bioavailability (rBA) studies in healthy volunteers. Consideration of WSV highlighted the use of rBA and BE studies for assigning variability, with some discussion of how bottom-up propagation of absorption variables contrasted with top-down assignment of variability using observed data. Another challenge is the formulation of non-BE batches, which can often be due to unplanned formulation or stability changes rather than deliberate design, and thus are no longer representative of the formulation design space, thereby impacting the safe space application. It was suggested that the use of sparse PK data in steady-state patient studies could be useful supplementary data for enhancing safe space robustness where data are limited. Concerns were raised for rare disease products due to limited data availability, questioning the regulatory application of PBBM for such conditions and potential impacts on safe space definition. Finally, the regulatory experience of applying PK-PD relationships to support safe space boundaries was limited; however, it was viewed as promising for justifying specification changes and safe space definitions.

Breakout session 3 considered the opportunities and challenges for developing a dissolution safe space for BCS II/IV drugs using PBBM. Participants discussed the acceptability of replacing f2 dissolution comparability in favour of a dissolution safe space for post-approval changes. There was broad agreement, provided that appropriate PBBM development and validation are aligned to the QoI and CoU, although passing f2 remains the current regulatory standard. If f2 is not met, PBBM could provide additional evidence to demonstrate an understanding of in vivo drug performance. PBBM should demonstrate the link from in vitro biopredictive dissolution to in vivo performance. With BCS II and IV drugs, particular consideration should be given to the use of non-relevant surfactants and over-discrimination in QC dissolution methods. The group proceeded to discuss the definition of CBAs for PBBM validation and which to prioritise in clinical testing. Companies commonly identify pCBAs based on the DS and drug product platform, then investigate using biorelevant and QC dissolution to rank their impact. Attributes with the largest effect may warrant clinical rBA testing. Final selection of CBAs should reflect the company’s concerns and specification setting whilst considering regulatory flexibility. Analysis should account for attribute (univariate vs. multivariate) type and placement within the drug product control space, including interactions between factors and global sensitivity analysis. PBBMs should incorporate drug physicochemical properties, drug product, and physiological variability using biorelevant dissolution methods to evaluate overall risk. Finally, the interplay of solubility, dissolution, precipitation and permeability for BCS II/IV drugs was discussed, with the key theme being understanding supersaturation levels relative to permeability, particularly whether such levels are reached for the given dose. Critical aspects highlighted further understanding of the drug–micelle interactions and impacts on solubility and ionisation. If supersaturation occurs, nucleation growth and precipitation kinetics need further exploration. PBBMs are crucial for modelling the interplay between the unbound fraction, dissolution, and permeability. Clinical data, alongside advanced gastrointestinal simulation models, can help to investigate supersaturation kinetics relative to dose and pH and provide key information on the most impactful processes for specific DSs or DPs. The role of P-gp on permeability should also be considered.

## 5. Meeting Conclusions

The two-day workshop provided opportunities for industrial, academic, and regulatory scientists and software companies to further discuss the latest developments in PBBM and to debate key topics relevant to the establishment of best practices and improved implementation. On Day 1, the meeting heard from industrial scientists through case study examples on how companies use PBBM in their decision making (early development, late development and regulatory specifications), and from regulatory scientists on PBBM within MIDD and how one could see both guidance and interactions evolving in the future. Three PBBM case studies were discussed with the aim of sharing the modeller’s insights and challenges on their respective experiences of PBBM development, validation and intended use. Each breakout group discussed the case study through the evaluation of three key questions. It is worth noting that the three case studies were developed at the same time as the company’s project questions, and the knowledge within the PBBM field has moved on considerably since then (e.g., through the incorporation of dissolution data, VBE, introduction of QoI, CoU); therefore, the groups also discussed how aspects could be considered based on current knowledge and best practices. An emerging theme from several of the talks was that the drug products discussed had lower quality risks than if they had only been assessed on the BCS class due to more thorough product development. On Day 2, the focus was on the PBBM regulatory angle, including regulatory experience with PBBM (MHRA/EMA) and the MMF approach (FDA), an industry-led approach to harmonising a PBBM report for regulatory submission, and the use of PBBM in setting clinically relevant dissolution specifications, including building a safe space.

Although it was clear from the presentations, discussions and BO sessions that we are making significant progress in the field of PBBM, both from an industry and regulatory perspective, the need to bring industry and regulators even closer together still exists. With the PBBM template publication, industry can adopt its use, so that regulators receive consistent material. Participating in regulatory meetings to share modelling strategies is also encouraged. From the regulators’ point of view, the use of PBBM is welcomed, and it is clear that a cross-functional team of experts is critical for its evaluation. Appropriate training is required for all assessors so that feedback on the intended use of the PBBM, together with the content of the report, can be discussed. Evolution of guidelines (ICH M15 MIDD, EMA concept paper) will certainly raise the opportunity for industry and global regulators to engage to reach a harmonised approach to the use of PBBM in defining drug product strategies and setting quality specifications for the ultimate benefit of patients.

## Figures and Tables

**Table 1:** Relative merits and challenges of Statement Pair 1.

Statement Pair	Review
All changes can be qualified without a BE study	Seems radical but SUPAC-IR and SUPAC-MR acknowledges Level 3 changes may be waived with IVIVC How do we know that the dissolution method is suitable for these changes? Dry granulation to wet granulation Soluble filler to insoluble filler When we think about this more, perhaps we are worried these changes will fundamentally affect the dissolution mechanism/characteristics of the product, such that any previous relationships are no longer valid
Only SUPAC-IR level 2	Conservative approach offers limited regulatory relief post approval—introduction already based on dissolution (fewer profiles). But more likely constrains changes to those that have previously been assessed. Or does it?

## Data Availability

No new data were created or analyzed in this study.

## Abbreviations

AAFE	Absolute Average Fold Error
AAM	Association for Accessible Medicines
AAPE	Absolute Average Prediction Error
AFMPS	Federal Agency for Medicines and Health Products (Belgium)
AI	Artificial Intelligence
API	Active Pharmaceutical Ingredient
ANDA	Abbreviated New Drug Application
ANVISA	Brazilian Health Regulatory Agency
APS	Academy of Pharmaceutical Sciences
AUC	Area Under the Curve
ARA	Acid-Reducing Agents
BE	Bioequivalence
BCS	Biopharmaceutics Classification System
BLA	Biologics Licence Application
BO	Breakout
Caco-2	Immortalised human colorectal adenocarcinoma cell line
CBA	Critical Bioavailability Attribute
CFD	Computational fluid dynamics
CMA	Critical Material Attribute
CMC	Chemistry, Manufacturing and Control management
CDER	Center for Drug Evaluation Research
Cmax	Maximum Concentration
CoU	Context of Use
CPP	Critical Process Parameters
CRDS	Clinically Relevant Dissolution Specifications
CQA	Critical Quality Attribute
CV	Coefficient of variation
DCS	Developability Classification System
DGM	Dynamic Gastric Model
DLM	Diffusion Layer Model
DMF	Drug Master File
DS	Drug Substance
DP	Drug Product
EFPIA	European Federation of Pharmaceutical Industry Associations
EMA	European Medicines Agency
ER	Extended Release
Fabs	Fraction absorbed
FAGG	Federal Agentschap voor Geneesmiddelen en Gezonheidsproducten
FDA	Food and Drug Administration
FeSSIF	Fed state simulated intestinal fluid
FiH	First In Human
FMEA	Failure Modes Effects Analysis
GI	Gastrointestinal
GMR	Geometric Mean Ratio
HA	Health Authority
HCL	Hydrochloric acid
HV	Healthy Volunteer
ICH	International Committee for Harmonisation
IHI	Innovative Health Initiative
IQ	International Consortium for Innovation and Quality in Pharmaceutical Development
IR	Immediate Release
IND	Investigational New Drug
IV	Intravenous
IVIVC	In Vitro In Vivo Correlation
IVIVR	In Vitro In Vivo Relationship
MAD	Maximum Absorbable Dose
M-CERSI	Maryland Center of Excellence in Regulatory Science and Innovation
MDCK	Madin–Darby Canine Kidney
MHRA	Medicines and Healthcare products Regulatory Agency
MIDD	Model Informed Drug Development
MIE	Model Integrated Evidence
ML	Machine Learning
MMF	Model Master File
MR	Modified Release
M&S	Modelling and simulation
MRI	Magnetic Resonance Imaging
NME	New Molecular Entity
NOMA	Norwegian Medical Products Agency
Non-BE	Non bioequivalent
NDA	New Drug Application
ODG	Office of Generic Drugs
OOS	Out Of Specification
pCBA	potential Critical Bioavailability Attribute
OrBiTo	Oral Biopharmaceutical Tools
PBBM	Physiologically Based Biopharmaceutics Model/Modelling
PBDT	Physiologically Based Dissolution Testing
PBPK	Physiologically Based Pharmacokinetics
Peff	Effective permeability
P-gP	P-glycoprotein
pH	Power of hydrogen (acidity or basicity measure)
PhRMA	Pharmaceutical Research and Manufacturers of America
PK	Pharmacokinetics
PMDA	Pharmaceuticals and Medical Devices Agency (Japan)
PK-PD	Pharmacokinetics–Pharmacodynamics
P/O	Predicted/observed
P-PSD	Product-specific Particle Size Distribution
PPI	Proton pump inhibitor
PSD	Particle Size Distribution
QA	Quality Attributes
QbD	Quality by design
QC	Quality Control
QD	Quaque Die (once daily dosing)
QoI	Question of Interest
QRA	Quality Risk Assessment
QSP	Quantitative Systems Pharmacology
rDCS	Refined Developability Classification System
rBA	Relative Bioavailability
RSD	Relative Standard Deviation
SA	Sensitivity Analysis
SAD	Single Ascending Dose
SOP	Standard Operating Procedure
SUPAC	Scale up, Post-Approval Changes
TIM	TNO Gastrointestinal Model
Tmax	Time to maximum concentration
VBE	Virtual Bioequivalence
V&V	Verification and validation
WSV	Within-Subject Variability

## References

[1] Tannergren C., Arora S., Babiskin A., Borges L., Chatterjee P., Cheng Y.-H., Dallmann A., Govada A., Heimbach T., Hingle M. (2025). Current State and New Horizons in Applications of Physiologically Based Biopharmaceutics Modeling (PBBM): A Workshop Report. Mol. Pharm..

[2] Mitra A., Suarez-Sharp S., Pepin X.J.H., Flanagan T., Zhao Y., Kotzagiorgis E., Parrott N., Sharan S., Tistaert C., Heimbach T. (2021). Applications of Physiologically Based Biopharmaceutics Modeling (PBBM) to Support Drug Product Quality: A Workshop Summary Report. J. Pharm. Sci..

[3] Ibrahim F. (2022). An Enabling Formulation of a Weakly Basic Compound Guided by Physiologically Based Biopharmaceutics Modeling (PBBM). J. Pharm. Sci..

[4] Vrenken P., Vertzoni M., Frechen S., Solodenko J., Meyer M., Muenster U., Dallmann A. (2025). Development of a Novel Physiologically Based Biopharmaceutics Modeling (PBBM) Framework Using the Open Systems Pharmacology Suite, Part 1: In Vitro Modeling of Vericiguat. Eur. J. Pharm. Sci..

[5] Wu D., Li M. (2023). Current State and Challenges of Physiologically Based Biopharmaceutics Modeling (PBBM) in Oral Drug Product Development. Pharm. Res..

[6] Mackie C., Arora S., Seo P., Moody R., Rege B., Pepin X., Heimbach T., Tannergren C., Mitra A., Suarez-Sharp S. (2024). Physiologically Based Biopharmaceutics Modeling (PBBM): Best Practices for Drug Product Quality, Regulatory and Industry Perspectives: 2023 Workshop Summary Report. Mol. Pharm..

[7] (2020). EMA. https://www.ema.europa.eu/en/documents/scientific-guideline/ich-m9-biopharmaceutics-classification-system-based-biowaivers-step-5_en.pdf.

[8] CDER (1997). Guidance for Industry SUPAC-MR: Modified Release Solid Oral Dosage Forms. https://www.fda.gov/media/70956/download.

[9] CDER (1995). Guidance for Industry: Immediate Release Solid Oral Dosage Forms Scale-Up and Postapproval Changes: Chemistry, Manufacturing, and Controls, In Vitro Dissolution Testing, and In Vivo Bioequivalence Documentation.

[10] (2022). Final Concept Paper M15: Model-Informed Drug Development General Principles Guideline.

[11] Arora S., Pepin X., Jamei M., Sharma P., Heimbach T., Wagner C., Bransford P., Kollipara S., Ahmed T., Hingle M. (2025). Development of a Physiologically Based Biopharmaceutics Model Report Template: Considerations for Improved Quality in View of Regulatory Submissions. Mol. Pharm..

[12] Abend A., Kuiper J., Hermans A., Kotwal P., Curran D., Lu X., Zhang L., Li H., Diaz D.A., Cohen M.J. (2019). Dissolution Testing in Drug Product Development: Workshop Summary Report. AAPS J..

[13] McAllister M., Flanagan T., Boon K., Pepin X., Tistaert C., Jamei M., Abend A., Kotzagiorgis E., Mackie C. (2020). Meeting Report: Developing Clinically Relevant Dissolution Specifications for Oral Drug Products—Industrial and Regulatory Perspectives. Pharmaceutics.

[14] Parrott N., Suarez-Sharp S., Kesisoglou F., Pathak S.M., Good D., Wagner C., Dallmann A., Mullin J., Patel N., Riedmaier A.E. (2021). Best Practices in the Development and Validation of Physiologically Based Biopharmaceutics Modeling. A Workshop Summary Report. J. Pharm. Sci..

[15] Pepin X.J.H., Dressman J., Parrott N., Delvadia P., Mitra A., Zhang X., Babiskin A., Kolhatkar V., Seo P., Taylor L.S. (2021). In Vitro Biopredictive Methods: A Workshop Summary Report. J. Pharm. Sci..

[16] Pepin X.J.H., Parrott N., Dressman J., Delvadia P., Mitra A., Zhang X., Babiskin A., Kolhatkar V., Suarez-Sharp S. (2021). Current State and Future Expectations of Translational Modeling Strategies to Support Drug Product Development, Manufacturing Changes and Controls: A Workshop Summary Report. J. Pharm. Sci..

[17] Heimbach T., Musuamba Tshinanu F., Raines K., Borges L., Kijima S., Malamatari M., Moody R., Veerasingham S., Seo P., Turner D. (2024). PBBM Considerations for Base Models, Model Validation, and Application Steps: Workshop Summary Report. Mol. Pharm..

[18] Pepin X., Arora S., Borges L., Cano-Vega M., Carducci T., Chatterjee P., Chen G., Cristofoletti R., Dallmann A., Delvadia P. (2024). Parameterization of Physiologically Based Biopharmaceutics Models: Workshop Summary Report. Mol. Pharm..

[19] EMA (2014). Guideline on the Pharmacokinetic and Clinical Evaluation of Modified Release Dosage Forms.

[20] EMA (2018). Guideline on the Reporting of Physiologically Based Pharmacokinetic (PBPK) Modelling and Simulation.

[21] FDA (2018). Physiologically Based Pharmacokinetic Analyses—Format and Content Guidance for Industry.

[22] FDA (2020). The Use of Physiologically Based Pharmacokinetic Analyses—Biopharmaceutics Applications for Oral Drug Product Development, Manufacturing Changes, and Controls. Guidance for Industry.

[23] Kuemmel C., Yang Y., Zhang X., Florian J., Zhu H., Tegenge M., Huang S.-M., Wang Y., Morrison T., Zineh I. (2020). Consideration of a Credibility Assessment Framework in Model-Informed Drug Development: Potential Application to Physiologically-Based Pharmacokinetic Modeling and Simulation. Pharmacomet. Syst. Pharmacol..

[24] ICH (2024). General Principles for Model-Informed Drug Development M15.

[25] Amidon G., Lennernäs H., Shah V., Crison J. (1995). A Theoretical Basis for a Biopharmaceutic Drug Classification: The Correlation of in Vitro Drug Product Dissolution and in Vivo Bioavailability. Pharm. Res..

[26] Butler J.M., Dressman J.B. (2010). The Developability Classification System: Application of Biopharmaceutics Concepts to Formulation Development. J. Pharm. Sci..

[27] Kesisoglou F., Chung J., Van Asperen J., Heimbach T. (2016). Physiologically Based Absorption Modeling to Impact Biopharmaceutics and Formulation Strategies in Drug Development—Industry Case Studies. J. Pharm. Sci..

[28] Heimbach T., Suarez-Sharp S., Kakhi M., Tsakalozou E., Seo P., Li M., Zhang X., Lin H.-P., Holmstock N., Olivares-Morales A. (2019). Dissolution and Translational Modeling Strategies Toward Establishing an In Vitro-In Vivo Link—A Workshop Summary Report. AAPS J..

[29] Sjögren E., Thörn H., Tannergren C. (2016). In Silico Modeling of Gastrointestinal Drug Absorption: Predictive Performance of Three Physiologically Based Absorption Models. Mol. Pharm..

[30] Tannergren C., Jadhav H., Eckernäs E., Fagerberg J., Augustijns P., Sjögren E. (2023). Physiologically Based Biopharmaceutics Modeling of Regional and Colon Absorption in Humans. Eur. J. Pharm. Biopharm..

[31] Stamatopoulos K., Ferrini P., Nguyen D., Zhang Y., Butler J.M., Hall J., Mistry N. (2023). Integrating In Vitro Biopharmaceutics into Physiologically Based Biopharmaceutic Model (PBBM) to Predict Food Effect of BCS IV Zwitterionic Drug (GSK3640254). Pharmaceutics.

[32] Johnson M., Pene Dumitrescu T., Joshi S.R., Mathew A., Bainbridge V., Zhan J., Lataillade M. (2022). Relative Bioavailability and Food Effect of GSK3640254 Tablet and Capsule Formulations in Healthy Participants. Clin. Pharmacol. Drug Dev..

[33] Pepin X.J.H., Flanagan T.R., Holt D.J., Eidelman A., Treacy D., Rowlings C.E. (2016). Justification of Drug Product Dissolution Rate and Drug Substance Particle Size Specifications Based on Absorption PBPK Modeling for Lesinurad Immediate Release Tablets. Mol. Pharm..

[34] Li M., Zhang X., Wu D., Anand O., Chen H., Raines K., Yu L. (2021). Understanding In Vivo Dissolution of Immediate Release (IR) Solid Oral Drug Products Containing Weak Acid BCS Class 2 (BCS Class 2a) Drugs. AAPS J..

[35] Schiller C., Frohlich C.-P., Giessmann T., Siegmund W., Monnikes H., Hosten N., Weitschies W. (2005). Intestinal Fluid Volumes and Transit of Dosage Forms as Assessed by Magnetic Resonance Imaging. Aliment. Pharmacol. Ther..

[36] Sutton S.C. (2009). Role of Physiological Intestinal Water in Oral Absorption. AAPS J..

[37] Sugano K., Nabuchi Y., Machida M., Aso Y. (2003). Prediction of Human Intestinal Permeability Using Artificial Membrane Permeability. Int. J. Pharm..

[38] Larhed A.W., Artursson P., Björk E. (1998). The Influence of Intestinal Mucus Components on the Diffusion of Drugs. Pharm. Res..

[39] Koenigsknecht M.J., Baker J.R., Wen B., Frances A., Zhang H., Yu A., Zhao T., Tsume Y., Pai M.P., Bleske B.E. (2017). In Vivo Dissolution and Systemic Absorption of Immediate Release Ibuprofen in Human Gastrointestinal Tract under Fed and Fasted Conditions. Mol. Pharm..

[40] Jamei M., Abrahamsson B., Brown J., Bevernage J., Bolger M.B., Heimbach T., Karlsson E., Kotzagiorgis E., Lindahl A., McAllister M. (2020). Current Status and Future Opportunities for Incorporation of Dissolution Data in PBPK Modeling for Pharmaceutical Development and Regulatory Applications: OrBiTo Consortium Commentary. Eur. J. Pharm. Biopharm..

[41] Ahmad A., Pepin X., Aarons L., Wang Y., Darwich A.S., Wood J.M., Tannergren C., Karlsson E., Patterson C., Thörn H. (2020). IMI—Oral Biopharmaceutics Tools Project—Evaluation of Bottom-up PBPK Prediction Success Part 4: Prediction Accuracy and Software Comparisons with Improved Data and Modelling Strategies. Eur. J. Pharm. Biopharm..

[42] Beran K., Hermans E., Holm R., Sepassi K., Dressman J. (2024). Using the Refined Developability Classification System (rDCS) to Guide the Design of Oral Formulations. J. Pharm. Sci..

[43] Beran K., Abrahamsson B., Charoo N., Cristofoletti R., Holm R., Kambayashi A., Langguth P., Parr A., Polli J.E., Shah V.P. (2024). Biowaiver Monographs for Immediate-Release Solid Oral Dosage Forms: Voriconazole. J. Pharm. Sci..

[44] Yu C. (2020). Evaluation of Gastric pH-Dependent Drug Interactions with Acid-Reducing Agents: Study Design, Data Analysis, and Clinical Implications Guidance for Industry.

[45] Dickinson P.A., Lee W.W., Stott P.W., Townsend A.I., Smart J.P., Ghahramani P., Hammett T., Billett L., Behn S., Gibb R.C. (2008). Clinical Relevance of Dissolution Testing in Quality by Design. AAPS J..

[46] Pepin X. Use of IVIVc and IVIVe to Support Formulation Development—Industrial Case Studies. Proceedings of the AAPS Meeting.

[47] Pepin X.J.H., Suarez-Sharp S. (2024). Effect of Food Composition on the PK of Isoniazid Quantitatively Explained Using Physiologically Based Biopharmaceutics Modeling. AAPS J..

[48] Pepin X., McAlpine V., Moir A., Mann J. (2023). Acalabrutinib Maleate Tablets: The Physiologically Based Biopharmaceutics Model behind the Drug Product Dissolution Specification. Mol. Pharm..

[49] Pepin X.J.H., Hynes S.M., Zahir H., Walker D., Semmens L.Q., Suarez-Sharp S. (2024). Understanding the Mechanisms of Food Effect on Omaveloxolone Pharmacokinetics through Physiologically Based Biopharmaceutics Modeling. CPT Pharmacomet. Syst. Pharmacol..

